# Chondroitin sulfate-modified tragacanth gum–gelatin composite nanocapsules loaded with curcumin nanocrystals for the treatment of arthritis

**DOI:** 10.1186/s12951-024-02540-2

**Published:** 2024-05-20

**Authors:** Junpeng Sun, Jiaqun Du, Xiaobang Liu, Jinyu An, Yu Hu, Jing Wang, Fu Zhu, Huicong Feng, Shuai Cheng, He Tian, Xifan Mei, Chao Wu

**Affiliations:** 1 Pharmacy School, Jinzhou Medical University, Jinzhou, Liaoning 121001 China; 2 Liaoning Provincial Collaborative Innovation Center of Medical Testing and Drug Development, Jinzhou Medical University, Jinzhou, Liaoning 121001 China; 3 School of Basic Medicine, Jinzhou Medical University, Jinzhou, Liaoning 121001 China; 4 The Third Affiliated Hospital of Jinzhou Medical University, Jinzhou, Liaoning 121001 China; 5 Liaoning Provincial Key Laboratory of Medical Tissue Engineering, Jinzhou Medical University, Jinzhou, Liaoning 121001 China

**Keywords:** Rheumatoid arthritis, Gouty arthritis, Curcumin, Tragacanth gum, Gelatin

## Abstract

Rheumatoid arthritis (RA) is a chronic autoimmune disease of yet undetermined etiology that is accompanied by significant oxidative stress, inflammatory responses,  and damage to joint tissues. In this study, we designed chondroitin sulfate (CS)-modified tragacanth gum–gelatin composite nanocapsules (CS-Cur-TGNCs) loaded with curcumin nanocrystals (Cur-NCs), which rely on the ability of CS to target CD44 to accumulate drugs in inflamed joints. Cur was encapsulated in the form of nanocrystals into tragacanth gum–gelatin composite nanocapsules (TGNCs) by using an inborn microcrystallization method, which produced CS-Cur-TGNCs with a particle size of approximately 80 ± 11.54 nm and a drug loading capacity of 54.18 ± 5.17%. In an in vitro drug release assay, CS-Cur-TGNCs showed MMP-2-responsive properties. During the treatment of RA, CS-Cur-TGNCs significantly inhibited oxidative stress, promoted the polarization of M2-type macrophages to M1-type macrophages, and decreased the expression of inflammatory factors (TNF-α, IL-1β, and IL-6). In addition, it also exerted excellent anti-inflammatory effects, and significantly alleviated the swelling of joints during the treatment of gouty arthritis (GA). Therefore, CS-Cur-TGNCs, as a novel drug delivery system, could lead to new ideas for clinical therapeutic regimens for RA and GA.

**Graphical Abstract:**

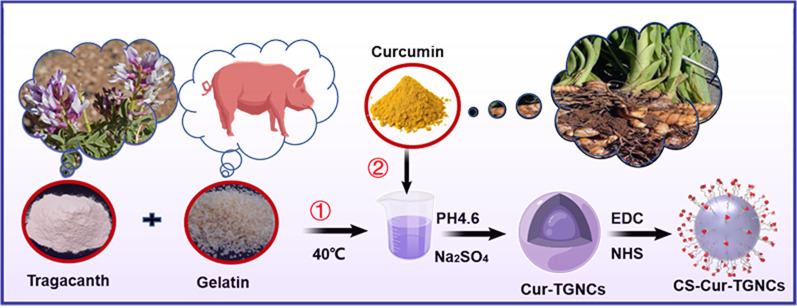

**Supplementary Information:**

The online version contains supplementary material available at 10.1186/s12951-024-02540-2.

## Introduction

Rheumatoid arthritis (RA) is a common chronic autoimmune disease characterized by swelling, pain, structural changes in the joints and dysfunction [1, 2]. Although the pathogenesis of RA is unclear, it is largely related to unwanted immune responses [3]. It has been extensively shown that innate immune cells, such as macrophages, monocytes and dendritic cells, are involved in the immune response to RA patients [4]. Macrophages are very abundant immune cells in the synovium and synovial fluid of RA patients [5]. An imbalance in the M1/M2 ratio at the site of disease results in an increase in the production of proinflammatory factors (IL-1β, iNOS, TNF-α, and IL-6) and matrix metalloproteinases (MMPs) by M1-type macrophages, which have been implicated in the pathogenesis of RA and can exacerbate joint damage [6, 7]. In contrast, M2-type macrophages are capable of releasing anti-inflammatory cytokines such as arginase-1 (Arg-1) and interleukin-10 (IL-10), which serve to inhibit inflammation and alleviate joint damage [8]. Synovial cells overgrow at sites of inflammation, leading to increased oxygen demand; thus, hypoxia also plays an important role in the pathogenesis of RA [9, 10]. Hypoxia at the disease site results in the upregulation of hypoxia-inducible factor-1α (HIF-1α) expression and the production of reactive oxygen species (ROS), which can damage biomolecules such as lipids, proteins, DNA, and mitochondria, resulting in damage to joint tissues [11]. It has been reported that when hypoxia is improved at the inflamed site, a reduction in ROS and downregulation of HIF-1α favor the conversion of M1-type macrophages to M2-type macrophages [12]. Therefore, reducing ROS, alleviating the hypoxic environment, inhibiting the secretion of proinflammatory factors, and promoting the polarization of M1-type macrophages to M2 macrophages are effective strategies for alleviating RA.

In traditional medicine, naturally sourced medicinal compounds are considered to have fewer side effects and lower toxicity, leading to their widespread use in nutritional supplements, pharmaceuticals, and drug delivery vehicles [13]. The commonly used natural compounds include tragacanth gum, gelatin, tannins and royal jelly [14, 15]. With the development of nanotechnology, gelatin and tragacanth gum have been widely used as drug delivery agents in the pharmaceutical field due to their unique properties, which provide new ideas for improving the therapeutic efficacy of these drugs [16, 17]. Gelatin is generated by degradation of the collagen portion of animal skin, bone and other tissues and is composed mainly of gelatin proteins but also contains small amounts of organic matter (nucleic acids, glycoproteins, cystine and their degradation products) and inorganic matter (salts and metal ions) [18]. Because of its good film-forming properties and safety, gelatin has been widely used in the pharmaceutical industry as a hard capsule shell, soft capsule shell, and microencapsulation wall material [19]. In addition, matrix metalloproteinases (MMPs), which are highly expressed in rheumatoid arthritis joints, also have gelatinase properties and are able to rapidly breakdown gelatin and promote gelatin capsule degradation [20]. Tragacanth gum has attracted the attention of researchers due to its anti-inflammatory and antioxidant effects [21, 22]. It is a dried secretion that is extracted from the branches and stems of Astragalus, and its soluble/insoluble constituents can be mixed with other polymers for the delivery of various drugs and bioactive ingredients [23]. Therefore, we used astragalus gum to functionalize gelatin capsules to prepare tragacanth gum–gelatin composite nanocapsules (TGNCs). As a novel nanodrug carrier, TGNCs not only have excellent anti-inflammatory and antioxidant activities but also enable site-directed release of drugs and enhances their therapeutic effects.

Currently, common drugs used clinically for the treatment of RA include nonsteroidal anti-inflammatory drugs (NSAIDs), disease-modifying anti-rheumatoid arthritis drugs (methotrexate, leflunomide, chloroquine and salazosulfapyridine), glucocorticoids (prednisone, dexamethasone), and biologics (abatacept) [24, 25]. Although these drugs are generally effective, their distribution at the site of inflamed joints is limited by their own physicochemical properties and the blood flow rate in joint tissues [26]. As a result, it is difficult to achieve high-concentration enrichment at the site of the disease, which can lead to low efficacy of the drug and a number of adverse effects, such as gastrointestinal disorders, renal failure, insulin resistance, cardiovascular disease and hypertension [27, 28]. Therefore, identifying safer, effective and economical drugs for treatment is essential. With the deepening of scientific research, many natural products with superior activity, such as curcumin, tripterygium glycosides and sinomenine, have attracted the attention of researchers [29, 30]. Among these products, curcumin (Cur) is widely used to treat immune-mediated inflammatory diseases because of its anti-inflammatory, antioxidant, and anti-aging effects [31]. However, its poor water solubility leads to low bioavailability, which seriously affects its clinical therapeutic efficacy [32].

Therefore, we synthesized tragacanth gum–gelatin composite nanocapsules loaded with curcumin nanocrystals (Cur-TGNCs) for the treatment of arthritis. However, the inability of Cur-TGNCs to actively target the site of joint inflammation limits the therapeutic efficacy of Cur in treating RA. To further improve the targeting efficacy of Cur-TGNCs, their surface was subjected to targeted modification to enhance their ability to aggregate at the site of joint inflammation and achieve targeted action [33]. A common approach is to modify peptides (monoclonal antibodies, cell-targeting peptides), folic acid, polysaccharides (chondroitin sulfate, dextran sulfate, mannose), nucleic acids (RNA nucleic acid aptamers, DNA nucleic acid aptamers), and others on the surface of the drug carrier [34, 35]. In contrast to other approaches, chondroitin sulfate (CS) acts as a polysaccharide that can protect articular cartilage while having a selective binding affinity for the CD44 receptor [36]. Inspired by these findings, we prepared CS-modified tragacanth gum–gelatin composite nanocapsules loaded with curcumin nanocrystals (CS-Cur-TGNCs) for arthritis-targeted therapy. The joint targeting ability and strong anti-inflammatory effects of CS-Cur-TGNCs in RA disease were verified by in vivo and in vitro experiments. In addition, gouty arthritis (GA) is a joint disease characterized by joint pain and inflammation, which are typical symptoms and are difficult to treat clinically due to its persistence and rapid onset. The joint-targeting ability and anti-inflammatory effects of CS-Cur-TGNCs in treating GA were further validated via in vitro and in vivo experiments. As a novel nanodelivery system, this approach provides new ideas for the clinical treatment of arthritis.

## Materials and methods

### Materials

Gelatin type B, tragacanth gum, Curcumin, Glacial acetic acid, Sodium sulfate, 37% formaldehyde, Chondroitin sulfate, EDC, NHS were purchased from Soleberg Reagent. Collagen (Type II, Chicken, Protein 60%, Yuanye), Fuchsin Complete Adjuvant (FCA), Fuchsin Incomplete Adjuvant (FICA) was purchased from Genye Reagent Company. Nitrendipine was obtained from Aladdin Chemical Reagent Co. CCK-8 kit, ROS kit, malondialdehyde (MDA) content assay kit, total glutathione (GSH-Px) content assay kit, superoxide dismutase (SOD) content assay kit and H&E kit were purchased from Beyotime Anti-HIF-1α, Anti-iNOS, anti-Arg-1, anti-IL-1β, anti-IL-10 were purchased from Abcam. RAW264.7 cells were obtained from Chinese Academy of Sciences.

### Preparation of Cur-TGNCs and CS-Cur-TGNCs

Accurately weighed 2.0 g of gelatin solid and 30 mg of tragacanth gum solid and dissolved in 100 mL of water at 40 °C in a water bath to obtain a 2% gelatin solution. About 8 mL of curcumin ethanol solution (2.5 mg/mL) was added dropwise into the above gelatin solution and stirred at 40 °C and 600 r/min for 3 min. The pH was adjusted to 4.26 with 10% glacial acetic acid and stirring was maintained. Using a constant-flow pump, add 5 ml of 20% sodium sulfate solution dropwise to the reaction solution, cool down to 15 °C in an ice bath, slowly add 0.3 mL of 37% formaldehyde, and continue stirring for 30 min. Centrifugation and drying yielded Cur-TGNCs nanopreparation. Precisely weighed chondroitin sulfate (CS) 30 mg, EDC(15 mg) and NHS (30 mg) were dissolved in 5 mL of triple-distilled water, and stirred at 25 °C for 12 h. Cur-TGNCs was added to the above solution, stirred for 12 h, centrifuged and dried to obtain CS-Cur-TGNCs. Subsequently, the drug loading was calculated at 425 nm using a UV spectrophotometer (UV-757CRT, Shanghai Precision Scientific Instrument Co., Ltd. Shanghai, China). The method was as follows:

Encapsulation efficiency (EE) = (total mass of Cur encapsulated in CS-Cur-TGNCs) / (total mass of CS-Cur-TGNCs) × 100%.

### Characterization of the CS-Cur-TGNCs

The morphology and structural features of CS-Cur-TGNCs were observed using a transmission electron microscope (JEM-1200EX; JEOL, Tokyo, Japan). The zeta potential and particle size of the prepared Cur-TGNCs and CS-Cur-TGNCs were measured with Zetasizer Nano ZS (Nano-ZS90, Malvern, UK). The crystalline state of curcumin was observed using a differential scanning thermal analyzer (DSC-60, Shimadzu, Kyoto, Japan). Drug crystal changes were measured by X-ray diffraction (PXRD, Rigaku Denki, Japan).

### ABTS radical cation-scavenging activity of CS-Cur-TGNCs

The CS-Cur-TGNCs were prepared at different concentrations (0, 12.5, 25, 50, 100, and 200 µg/mL), and UV‒VIS absorption spectra were obtained using an ABTS kit at different concentrations. Simulation of CS-Cur-TGNCs activity by catalase (CAT) and superoxide dismutase (SOD). Different concentrations of CS-Cur-TGNCs and H_2_O_2_ (100 µM) were reacted for 30 min, after which the supernatant was mixed with an equal volume of KI (1 M) and incubated for 5 min. Afterward, the UV‒VIS absorption spectra were collected.

### In vitro cell assay

#### Cell culture

RAW264.7 cells were obtained from the cell bank of the Chinese Academy of Sciences. DMEM (Gibco, Grand Island, NY, USA) containing 10% fetal bovine serum (FBS), penicillin and streptomycin (Gibco, Grand Island, NY, USA) was used and cultured at 37 °C and 5% CO_2_. 0.25% trypsin digestion was used during passaging. In addition, cells were frozen using FBS containing 10% DMSO.

#### Cellular uptake

The CS-Cur-TGNCs were incubated with rhodamine B. Next, RAW264.7 cells were inoculated and cultured in confocal dishes and incubated with LPS (1000 ng/mL) for 24 h. RAW264.7 cells were incubated with 20 µM Cur as well as Cur-TGNCs or CS-Cur-TGNCs for 2 h. Then, the cells were fixed with 4% paraformaldehyde for 30 min and washed three times with PBS, after which the nuclei were stained with DAPI for 30 min. Finally, images were obtained using a confocal microscope (N-SIM, Nikon, Tokyo, Japan).

#### Immunofluorescence staining analysis

RAW264.7 cells were inoculated into confocal dishes and incubated for 24 h under anoxic conditions with LPS (1000 ng/mL). The cells were then treated with different preparation agents (PBS, Cur, Cur-TGNC, or CS-Cur-TGNC) for an additional 24 h. Next, the cells were fixed with 4% paraformaldehyde for 30 min, incubated with 0.1% Triton X-100 for 15 min, and incubated overnight at 4 °C with primary antibodies (a rabbit monoclonal antibody against Inos, HIF-1α, IL-1β, Arg-1, or HIF-1α). The next day, the cells were incubated with secondary antibodies in the dark for 2 h. Subsequently, the nuclei were stained with DAPI for 15 min and observed under a confocal microscope (N-SIM, Nikon, Tokyo, Japan); images were obtained.

### In vivo experiment

#### Establishment and treatment of animal models

Six- to eight-week-old DBA/1 mice were purchased from Vital River (Beijing, China) and housed under adequate water, food and pathogen-free conditions. All animal experimental protocols were approved by the Laboratory Animal Ethics Committee of Jinzhou Medical University. A double immunization method was used to establish the CIA mouse model. The first immunization was completed by intradermal injection of an equal volume of chicken type II collagen solution (2 mg/mL) and an emulsion of complete Freund’s adjuvant (4 mg/mL) into the tail of the mice. After 21 days, the mice were booster immunized with chicken type II collagen solution emulsified in incomplete Freund’s adjuvant. The nanopreparation agent (10 mg/kg Cur) was injected into the tail vein every three days from Day 28 to Day 43. On Day 46, the mice were visually scored for joint inflammation, and the paws were scored under the following conditions: 4 = severe erythema and edema present in the ankles, feet, and toes, 3 = moderate erythema and edema extending from the metatarsophalangeal joints to the ankles, 2 = mild erythema and edema extending to the mid-foot and ankle joints, 1 = mild erythema and swelling confined to the ankle joints and mid-foot; and 0 = normal. Mice were weighed, and differences in body weight were recorded. The thickness of the ankle joint of the hind paw was recorded using Vernier calipers, and the position of each measurement was kept consistent. In addition, the distance traveled, speed and resting time of the different groups of mice were measured every 10 min for 30 min to assess the therapeutic effect of the different preparations on the RA mice.

#### Establishment and treatment of the GA rat model

Synthesis of MSU: Uric acid (2 g) was dissolved in 400 mL of water (70 °C) containing 12 mL of 1MNaOH, the pH of the solution was adjusted to 7.1–7.2, and the solution was slowly stirred at room temperature overnight at 4 °C. The next day, the precipitate was separated, dried, and filtered through a 250 μm metal mesh, and the MSU was sterilized using UV light irradiation.

Adult SD rats (10–12 weeks, 180–220 g) were purchased from Jinzhou Medical University. All animal experimental protocols were approved by the Animal Ethics Committee of Jinzhou Medical University. SD rats were housed under adequate water, food and pathogen-free conditions. After the animals were acclimatized to the environment, they were anesthetized with 1% sodium pentobarbital (50 mg/kg) and injected with MSU suspension (1.25 mg/100 µL) in the ankle joint. Rats were pretreated with PBS, Cur, Cur-TGNCs or CS-Cur-TGNCs (equivalent to 10 mg/kg Cur) 1 h before MSU injection. The diameter of the rat ankle joints was measured and photographed after 24 h using Vernier calipers. The distance traveled, speed and resting time of the different groups of mice were also measured every 10 min for 30 min to assess the therapeutic effect of the different preparations on the GA rats. Joint dysfunction and inflammation were visually scored. The inflammation indices were as follows: Grade 0, normal; Grade 1, visible erythema, mild swelling and bone signs; Grade 2, significant joint erythema and bone signs disappeared, but swelling was limited to the joints; Grade 3, swelling of the limb outside the left joint. The scores for each rat were summarized, and the highest score was 16.

#### q‒PCR analysis

Ankle tissues were collected from DBA/1 mice and SD rats. Joint tissues were repeatedly frozen-thawed using liquid nitrogen and then ground in TRIzol at 4 °C. Total RNA was subsequently obtained by extraction, purification, and drying. After testing the RNA concentration of the samples, cDNA was obtained by reverse transcription using a reverse transcription kit (PrimeScriptTM RT Master Mix, TaKaRa, Japan) according to the manufacturer’s standard protocol. qRT‒PCR was carried out with SYBR Green real-time PCR Master Mix on a LightCycler 96 instrument. β-Actin was used as an internal reference. The specific sequences of the primers used are shown in Table 1 (rat) and Table 2 (mouse).

**Table 1:** List of primer sequences

Gene	Forward primer (5 − 3)		Reverse primer (3’-5’)
IL-6		TCCTACCCCAACTTCCAATGC	GGTTTGCCGAGTAGACCTCAT
TNF-α		AGATGTGGAACTGGCAGAGG	CACGAGCAGGAATGAGAAGAG
IL-1β		GACAGAACATAAGCCAACAAGTC	ACACAGGACAGGTATAGATTCTTC
β-actin		CACCCGCGAGTACAACCTTC	CCCATACCCACCATCACACC

**Table 2:** List of primer sequences

Gene	Forward primer (5 − 3)		Reverse primer (3’-5’)
IL-6		TTCCATCCAGTTGCCTTCTTG	AATTAAGCCTCCGACTTGTGAA
iNOS		GGCTGTCACGGAGATCAATG	TGGTAGTAGTAGAATGGAGATAGGA
TNF-α		CACCACGCTCTTCTGTCTAC	GGCTACAGGCTTGTCACTC
IL-1β		ATCTCGCAGCAGCACATCA	CCAGCAGGTTATCATCATCATCC
β-actin		CTATTGGCAACGCGCGGTTC	ACTGTGTTGGCATAGAGGTCTT

### Statistical analysis

All the data were analyzed using GraphPad (version 8.0) and are expressed as the mean ± standard deviation (SD). The data were analyzed using one-way analysis of variance (ANOVA) to compare the differences between groups. When the variance was not equal, the Kruskal‒Wallis test was used. When the P value was less than 0.05, it was considered to be statistically significant.

## Results

### Preparation and characterization of CS-Cur-TGNCs

**Fig. 1:**
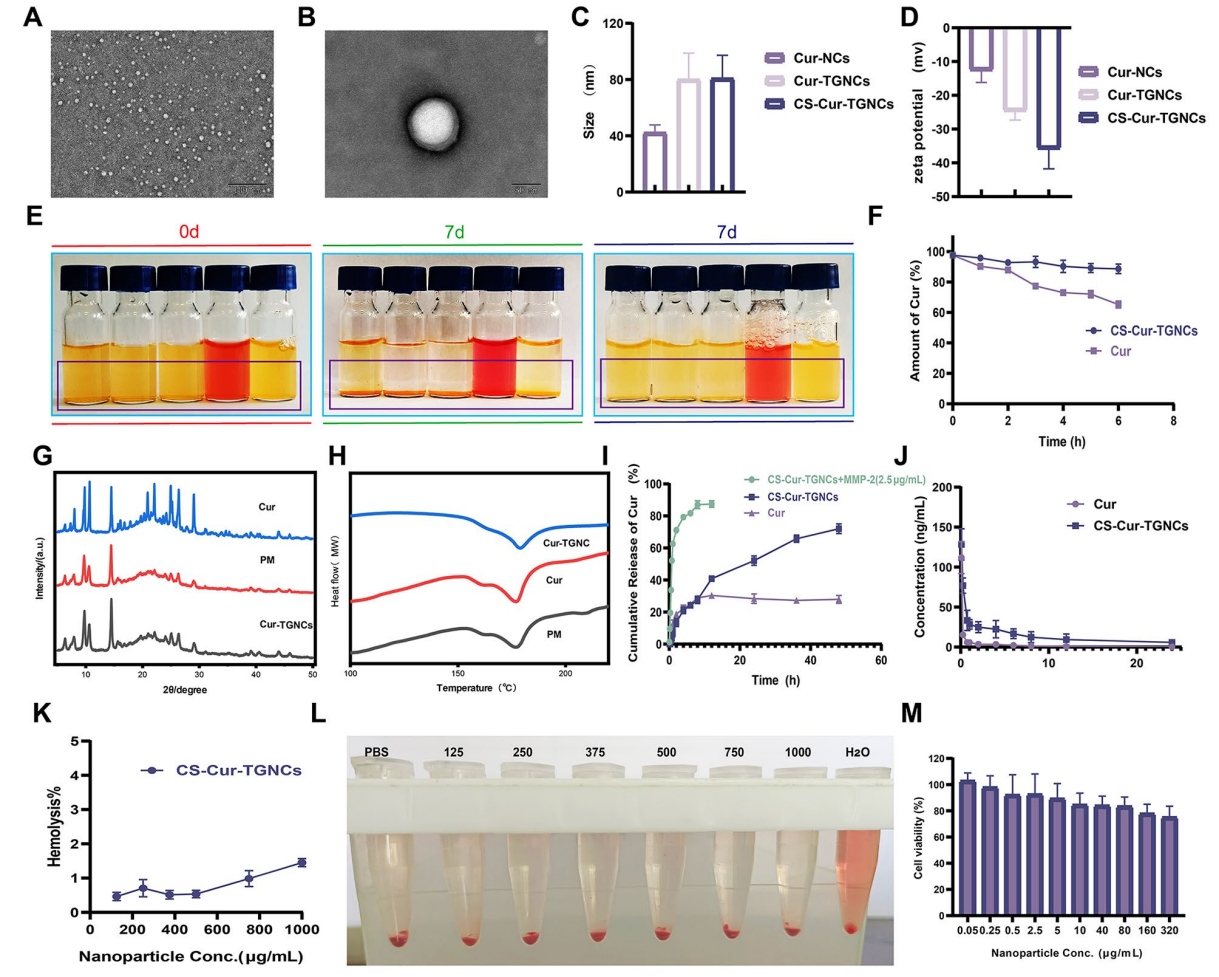
Preparation and characterization of CS-Cur-TGNCs. (**A**) The TEM imaging of CS-Cur-TGNCs. (**B**) The magnified TEM imaging of CS-Cur-TGNCs. (**C**) The particle size of Cur-NCs, Cur-TGNCs and CS-Cur-TGNCs. (**D**) The zeta potential of Cur-NCs, Cur-TGNCs and CS-Cur-TGNCs. (**E**) Images of CS-Cur-TGNCs in different buffers at 0 d and 7 d (from left to right, water, PBS, 0.9% NaCl, DMEM, and 10% FBS) as well as resuspension after 7 d (right panel). (**F**) Cur Variation of concentration over time. (**G**) The PXRD patterns of Cur, Cur-TGNCs and PM (physical mixture of Cur, tragacanth gum and gelatin). (**H**) The DSC spectra of Cur, Cur-TGNCs and PM (physical mixture of Cur, tragacanth gum and gelatin). (**I**) In vivo drug release curves of Cur and CS-Cur-TGNCs. (**J**) The plasma concentration-time curve of Cur and CS-Cur-TGNCs. (**K**, **I**) The hemolysis experiment diagram and hemolysis rate of CS-Cur-TGNCs. (**M**) The cytotoxicity of CS-Cur-TGNCs on RAW264.7 cells

TEM is a means of observing the morphological structure of nanoparticles. Figure 1A and B show that CS-Cur-TGNCs were regular monodisperse solid spheres approximately 80 nm in size, with a distinct core-shell structure and an inner core approximately 75 nm in size and an outer capsule wall material thickness of approximately 5 nm. DLS revealed that the sizes of the Cur-NCs, Cur-TGNCs and CS-Cur-TGNCs were 42.29 ± 5.42 nm (PDI: 0.232 ± 0.071), 80.05 ± 18.7 nm (PDI: 0.301 ± 0.054) and 80.80 ± 16.47 nm (PDI: 0.337 ± 0.049), respectively, with zeta potentials of -12.66 ± 3.52 mv, -24.70 ± 2.61 mv and − 35.87 ± 5.90 mv, respectively (Fig. 1C and D and S1). Compared with Cur-TGNCs, CS-Cur-TGNCs exhibited a slight increase in particle size and a significant decrease in potential, which was related to the strong negative charge of CS. FTIR spectroscopy was used to further verify whether CS was successfully grafted on the surface of Cur-TGNCs, and the results were shown in Figure S2. The stretching vibration of the lipid sulfate group (S = O) was present at 1020 cm^− 1^ for CS, while the characteristic peak of CS was absent for Cur-TGNCs. Compared with Cur-TGNCs, CS-Cur-TGNCs showed the lipid sulfate group and C-O stretching vibration of CS at 1205 cm^− 1^ and 1029 cm^− 1^, which indicated that CS had been successfully modified on the surface of Cur-TGNCs. Subsequently, we observed the stability of CS-Cur-TGNCs in different physiological solutions (water, PBS, 0.9% NaCl, DMEM, and 10% FBS). The results in Fig. 1E showed that CS-Cur-TGNCs had good dispersion in all media. After 7 d, CS-Cur-TGNCs exhibited the phenomenon of sedimentation in all solutions, and it was able to disperse uniformly after uniform shaking, indicating its excellent dispersibility. The changes in particle size, potential and PDI values were detected at 0 d and 7 d, and the results showed that the CS-Cur-TGNCs in different media did not change significantly within seven days and exhibited good stability (Fig. S3). The stability of Cur was poor, so the change in the stability of Cur in the CS-Cur-TGNCs was assessed via drug stability tests. The degradation rates of CS-Cur-TGNCs and free Cur in PBS (37 °C, pH = 7.4) after 6 h were approximately 15% and 40%, respectively, and the degradation rate of Cur in the CS-Cur-TGNCs was 0.38 times greater than that in the free Cur (Fig. 1F). The CS-Cur-TGNCs significantly improved the stability of Cur. The crystallinity and thermal properties of Cur, Cur-TGNCs and PM were observed by PXRD and DSC. The PXRD curves showed that Cur is a crystalline drug with multiple diffraction peaks, and strong crystal diffraction peaks appeared in the range of 3° to 50°. The positions of the characteristic diffraction peaks of Cur, PM and Cur-TGNCs did not change significantly, indicating that Cur existed in a nanocrystalline form in Cur-TGNCs. Compared with that of Cur, the intensity of the diffraction peak of Cur-TGNCs was significantly lower (Fig. 1G). The crystallinities of Cur and Cur-TGNCs were calculated to be 52.82% and 36.59%, respectively, using Origin software (originpro 2023). The crystallinity of Cur was significantly lower in Cur-TGNCs than in other materials, and drugs with low crystallinity were more favorable for dissolution and absorption. This result was further verified by the DSC results (Fig. 1H). The characteristic absorption peaks of the Cur, PM and Cur-TGNC samples appeared near 176 °C, and compared to those of Cur, the intensities of the absorption peaks of Cur-TGNCs were significantly lower. This is related to the fact that Cur exists in the form of nanocrystals in Cur-TGNCs. According to the Ostwald freundlich equation and Noyes-Whitney equation, the smaller particle size and larger specific surface area of Cur nanocrystals can increase the solubility and dissolution rate of Cur, thereby improving the bioavailability and efficacy of the Cur. Next, we determined the in vitro release of Cur from the CS-Cur-TGNCs. As shown in Fig. 1I, in the absence of MMP-2, 40% of the CS-Cur-TGNCs were released after 12 h, indicating that the release of these compounds was significantly slowed. In the presence of MMP-2, 90% of the CS-Cur-TGNCs were released after 12 h. This finding suggested that CS-Cur-TGNCs are able to rapidly release Cur in the inflamed joints of RA patients (in which MMPs are highly expressed), which in turn increases drug bioavailability and improves therapeutic efficacy. This finding is supported by the results of pharmacokinetic experiments; the t_1/2_ values of Cur and CS-Cur-TGNCs were 0.06 ± 0.01 h and 0.40 ± 0.15 h, respectively. The AUC0-t values of Cur and CS-Cur-TGNCs were 56.24 ± 12.97 ng/L*h and 344.49 ± 147.37 ng/L*h, respectively. Compared with Cur, CS-Cur-TGNCs had approximately 7-fold and 6-fold greater t_1/2_ and AUC0-t values, respectively (Fig. 1J). These findings indicated that CS-Cur-TGNCs significantly improved the bioavailability of Cur. Hemolysis and cytotoxicity assays were used to study the biosafety of the CS-Cur-TGNCs. The results of the hemolysis assay showed that the CS-Cur-TGNCs did not exhibit hemolysis in the range of 125–1000 µg/mL (Fig. 1K, L). CCK-8 assays revealed that the viability of RAW264.7 cells cultured in the presence of 80 µg/mL CS-Cur-TGNC was greater than 80% (Fig. 1M). The results of hemolysis and cytotoxicity assays indicated that the CS-Cur-TGNCs had good biosafety. Based on these results, we believe that CS-Cur-TGNCs have potential as a novel drug delivery system for the treatment of arthritis.

### Cell uptake and targeting ability of the CS-Cur-TGNCs

**Fig. 2:**
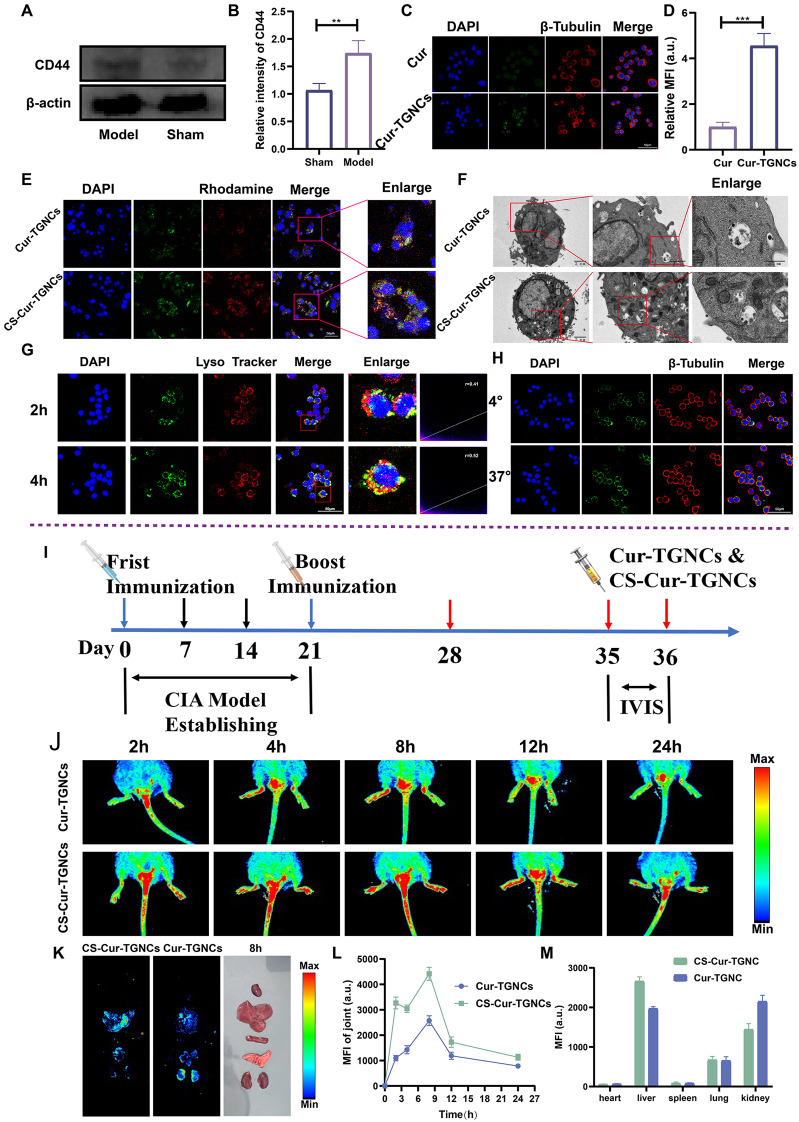
Cell uptake and targeting validation of CS-Cur-TGNCs. (**A**, **B**) Expression and quantification of CD44 in the joints of healthy mice and CIA mice. (**C**, **D**) The uptake of Cur and Cur-TGNCs by RAW264.7 cells after inflammatory activation and quantitative statistics. (**E**) The uptake of Cur-TGNC and CS-Cur-TGNCs by RAW264.7 cells after inflammatory activation and quantitative statistics. (**F**) Cell morphology and mitochondrial morphology of each group under TEM; Sclar bar = 2 μm, 1 μm and 500 nm. (**G**) RAW264.7 on the uptake of CS-Cur-TGNCs at various points in time. Red light (lysosomal probe). (**H**) RAW264.7 uptake of CS-Cur-TGNCs at different temperatures at 2 h. (**I**) Experimental outline. (**J**, **L**) Pictures and statistical analysis of the mouse IVIS system. (**K**, **M**) Pictorial and statistical analysis of the vital metabolic organs of the mouse IVIS system. Data are shown as the mean ± SD (*n* = 3). *P < 0.05, **P < 0.01, ***P < 0.001

CD44 expression at the junction sites was detected via Western blot experiments, and the ability of CS-Cur-TGNC to target cells and tissues was assessed via cellular uptake analysis and in vivo imaging systems. Western blot analysis (Fig. 2A, B) revealed a significant increase in CD44 expression in the joint area of CIA mice compared with that in the sham group (approximately 1.7-fold). CLSM was applied to determine the ability of the RAW264.7 cells to take up free Cur, Cur-TGNCs and CS-Cur-TGNCs. As shown in Fig. 2C, the intensity of uptake of Cur-TGNCs by RAW264.7 cells was significantly greater than that of Cur, and the fluorescence level was approximately 4.5 times greater than that of Cur (Fig. 2D). As shown in Fig. 2E, the fluorescence of CS-Cur-TGNCs was greater than that of Cur-TGNCs, indicating that RAW264.7 cells had a greater uptake effect on the CS-Cur-TGNCs. This is because the sulfated groups of CS can interact with the CD44 receptor on the surface of macrophages, and the negative charge of the sulfated groups and the amino glucose units can also stabilize the interaction between CS and CD44 [37, 38]. Moreover, Cur (green light) exhibited a high degree of consistency with Cur-TGNCs and CS-Cur-TGNCs (red light), suggesting that the increase in Cur absorption is closely related to the structural properties of the CS-Cur-TGNCs. The same phenomenon was observed using TEM (Fig. 2F). CS-Cur-TGNCs and Cur-TGNCs were taken up by activated RAW264.7 cells mainly by endocytosis. Due to the ability of CS to target CD44, RAW264.7 cells exhibited increased uptake of CS-Cur-TGNCs. More CS-Cur-TGNC nanoparticles were observed inside the cells. We next used lysosomal staining to assess the intracellular localization of the CS-Cur-TGNCs in RAW264.7 cells. CS-Cur-TGNCs (green) colocalized well with LysoTracker (red) (Fig. 2G). The colocalization coefficients at 2 h and 4 h were 0.41 and 0.52, respectively. Therefore, we inferred that the lysosomal structure within RAW264.7 cells mediates the intracellular internalization of CS-Cur-TGNCs, which is a common phenomenon in the cellular uptake of nanoparticles. The effect of energy on the uptake of the CS-Cur-TGNCs by RAW264.7 cells was explored by performing cell uptake experiments at 4 °C and 37 °C. The results showed that the intensity of CS-Cur-TGNC uptake by RAW264.7 cells was significantly greater at 37 °C than at 4 °C (Fig. 2H). These findings indicate that the RAW264.7 cell-mediated cellular internalization of CS-Cur-TGNCs was energy dependent. To evaluate the joint targeting ability of the CS-Cur-TGNCs, as shown in Fig. 2I, we established a CIA mouse model using the repeat immunization method. At 35 days, joint images of the mice at different time points were obtained using an in vivo imaging system (IVIS). The results showed that Cur-TGNCs were able to accumulate at the joint site. Compared with that of Cur-TGNCs, the fluorescence intensity of CS-Cur-TGNCs was significantly greater, with the highest fluorescence occurring at 8 h (Fig. 2G and L). It has been well demonstrated that the use of CS-Cur-TGNCs as a nanodelivery system for targeting joints can achieve accumulation at the joint site, which will improve the therapeutic efficacy of Cur. In addition, among the important metabolic organs (heart, liver, spleen, lungs, and kidneys) in mice, the liver and kidneys exhibited increased fluorescence intensity (Fig. 2K and M). These results indicated that the CS-Cur-TGNCs were metabolized mainly by the liver. This is similar to the metabolic phenomenon of most nanoparticles [39]. The liver as main metabolic organ can metabolism and clear the foreign substances via the process of internalization by hepatic cells such as Kupffer cells and liver endothelial cells. These results suggest that CS-Cur-TGNCs can be taken up by macrophages and accumulate at the joint site.

### Exploring the mechanism of action of Cur in rheumatoid arthritis

**Fig. 3:**
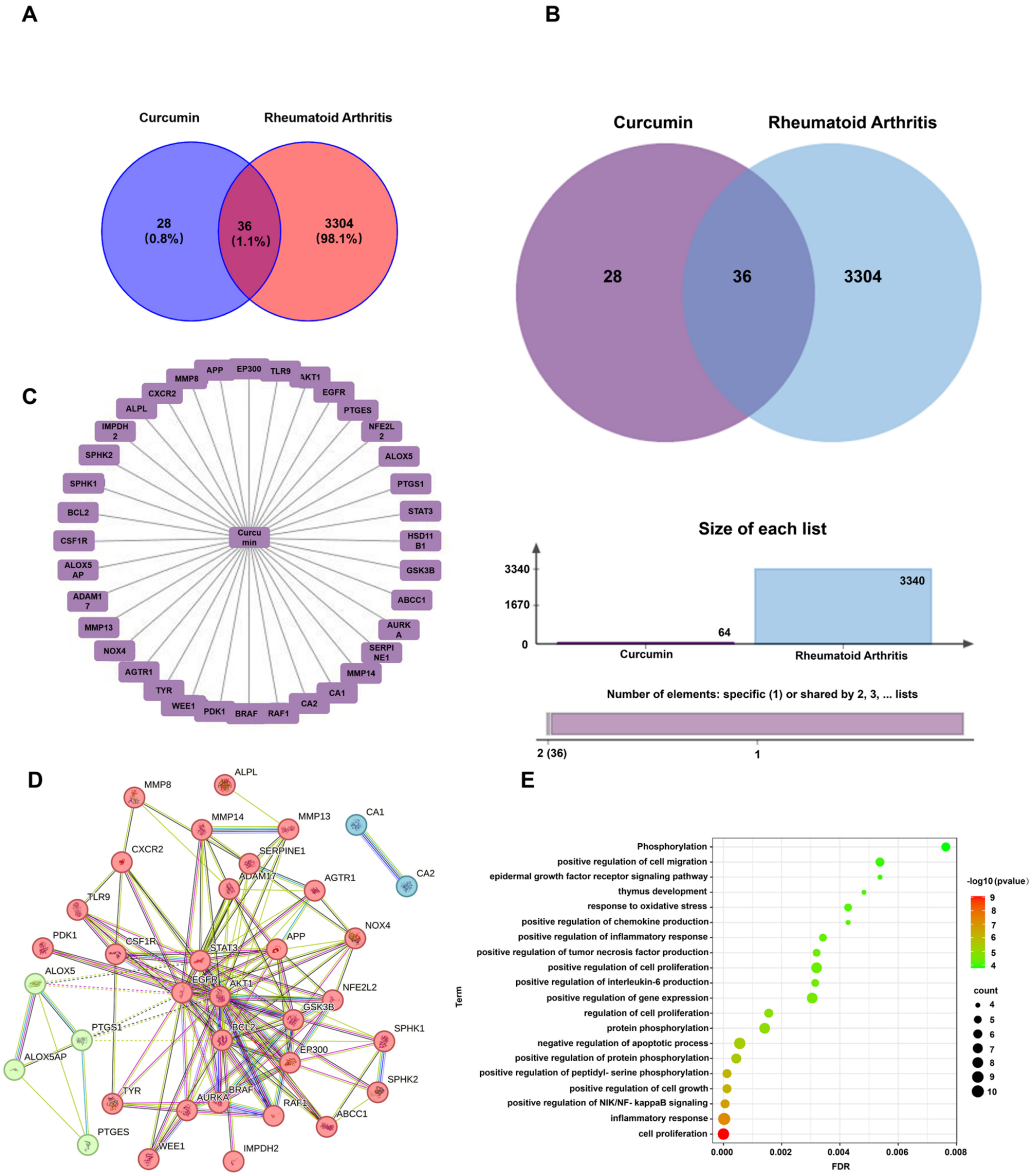
Exploring the mechanism of action of Cur in rheumatoid arthritis. (**A**) and (**B**) Venn diagrams and crossover plots of active ingredients for Cur and RA disease targets. (**C**) Network plot of Cur-RA target interactions. (**D**) PPI network plots of crossover targets in the STRING database. (**E**) Target GO/KEGG enrichment analysis plot

To clarify the disease targets of Cur, we performed target prediction with the Swiss Target Prediction Database, and the prediction results were collated to obtain 64 drug targets. For RA disease-related targets, the DisGeNET and GeneCards databases were used to collate the predicted relevant targets with probable values greater than 2.5, from which 3340 RA disease-related targets were obtained. Cross-analysis of disease targets and predicted drug targets yielded 36 intersecting targets (Fig. 3A and B). To visualize the interactions between Cur and its targets, cross-targets and Cur were imported into Cytoscape 3.8.2 software to construct a component-disease target interaction network (Fig. 3C). To analyze the target interactions, 36 intersecting targets were imported into STRING, and “*Homo sapiens*” was selected as the “Species” to construct a PPI network (Fig. 3D), which consisted of a total of 36 nodes (representing the interacting targets) and 138 edges (representing the interaction relationships between the targets). As shown in the figure, the core targets we obtained were mainly ALPLCA1, MMP8, ABCC1, ADAM17, AGTR1, AKT1, APPAURKA, BCL2, BRAF, CSF1R, CXCR2, EGFR and other genes, which implies that these targets play important roles in the network. GO functional annotation of the core targets of curcumin in RA was performed, and the results are shown in Fig. 3E. Larger bubbles represent more genes enriched in that GO entry, and redder bubbles indicate more significant GO features. The obtained GO-enriched terms were enriched mainly in the terms “positive regulation of peptidyl-serine phosphorylation”, “positive regulation of cell growth”, “positive regulation of NIK/NF-κB signaling”, “inflammatory response” and “response to oxidative stress”. In this study, we used network pharmacology to explore the molecular mechanism of Cur in the treatment of RA and to provide a reference for subsequent studies.

### Antioxidant stress activity of the CS-Cur-TGNCs

**Fig. 4:**
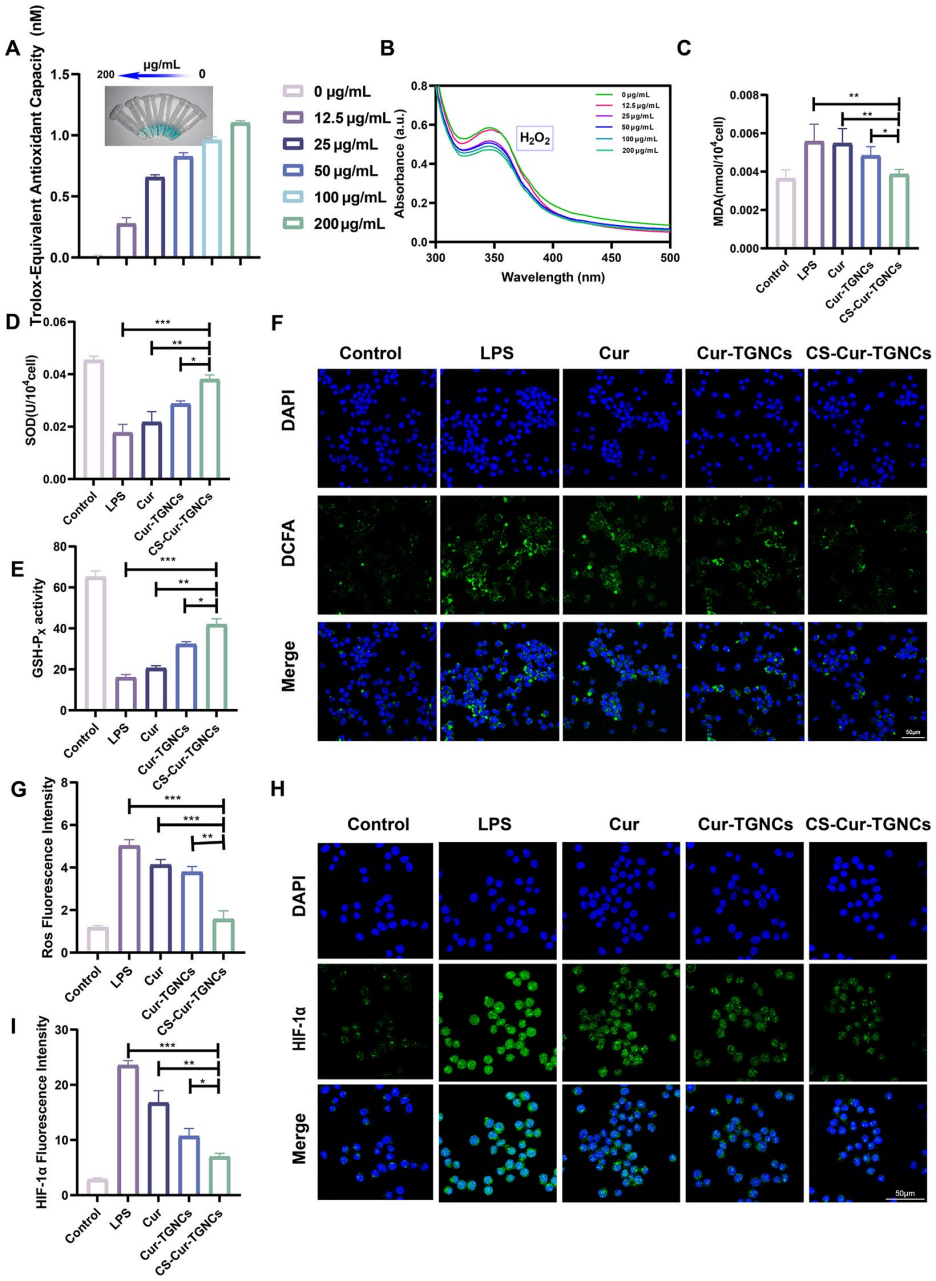
Antioxidant stress activity of CS-Cur-TGNCs. (**A**) ROS scavenging ability of CS-Cur-TGNCs at different concentrations after reacting with ABTS solution. (**B**) The UV-Vis spectra of I2/I3- after KI were incubated with different concentrations of CS-Cur-TGNCs for 30 min. (**C**–**E**) MDA and SOD concentrations and GSH activity in different groups of RAW264.7 cells. (**F**, **G**) DCFH-DA probe fluorescence Images and quantitative analysis of different groups of RAW264.7 cells. (**H**, **I**) HIF-α fluorescence images and quantitative analysis of different groups of RAW264.7 cells. Data are shown as the mean ± SD (*n* = 3). *P < 0.05, **P < 0.01, ***P < 0.001

The antioxidant effect of CS-Cur-TGNCs was detected using an oxidative stress-related kit (ABTS kit, KI reagent, MDA kit, SOD kit and GSH kit) and cellular immunofluorescence. The ABTS assay is often used to detect the in vitro antioxidant capacity of substances. When ABTS is oxidized to a stable blue‒green ABTS + radical, there is a characteristic absorption peak at 405 nm. The presence of an antioxidant inhibits the production of ABTS+, resulting in a lighter color and lower absorbance of the solution. Figure 4A shows that at concentrations of CS-Cur-TGNCs ranging from 0 to 200 µg/mL, the color of the solution gradually changed from blue‒green to light blue‒green, and the absorbance values also changed significantly. These results indicated that the ROS scavenging ability of the CS-Cur-TGNCs was concentration-dependent. As the concentration of CS-Cur-TGNCs increased, the absorption intensity of I2/I3^−^ at 350 nm gradually decreased. The lowest absorption density was observed at a CS-Cur-TGNCs concentration of 200 µg/mL, which further indicated that the scavenging effect of CS-Cur-TGNCs on H_2_O_2_ was significantly concentration dependent (Fig. 4B). Next, we examined the antioxidant effects of CS-Cur-TGNCs at the cellular level using MDA, SOD, and GSH assays. Among the groups, the RAW264.7 cells had the highest level of MDA after inflammatory activation and the lowest level of MDA in the CS-Cur-TGNCs group; these values were approximately 0.56-fold greater than those in the LPS group (Fig. 4C). In addition, the levels of SOD and GSH were the lowest after LPS stimulation, and the levels gradually increased in the different treatment groups, with the highest levels occurring in the CS-Cur-TGNCs group (Fig. 4D, E). These results again demonstrated that CS-Cur-TGNCs have excellent antioxidative stress effects. Cellular fluorescence experiments using ROS fluorescent probes were used to evaluate the antioxidant activity of the CS-Cur-TGNCs. The results showed that the highest fluorescence intensity was observed in the LPS-induced group, and the fluorescence intensity gradually decreased in the Cur, Cur-TGNCs and CS-Cur-TGNCs groups. Among the groups, the CS-Cur-TGNCs group had the weakest fluorescence signal, indicating that the CS-Cur-TGNCs had a strong ability to reduce ROS levels (Fig. 4F, G). HIF-1α is a key indicator of oxidative stress, and changes in HIF-1α expression reflect the level of oxidative stress. We assessed the expression of HIF-1α in cells by cytofluorimetric assay. The results showed that the upregulated HIF-1α in RAW264.7 cells was reduced by the Cur, Cur-TGNCs and CS-Cur-TGNCs treatments, especially in the case of the CS-Cur-TGNCs treatment, which had a stronger effect (Fig. 4H, I). These results suggest that CS-Cur-TGNCs can alleviate oxidative stress, ameliorate hypoxia at joint sites, and play a therapeutic role in the treatment of arthritic diseases.

### Anti-inflammatory activity of the CS-Cur-TGNCs

**Fig. 5:**
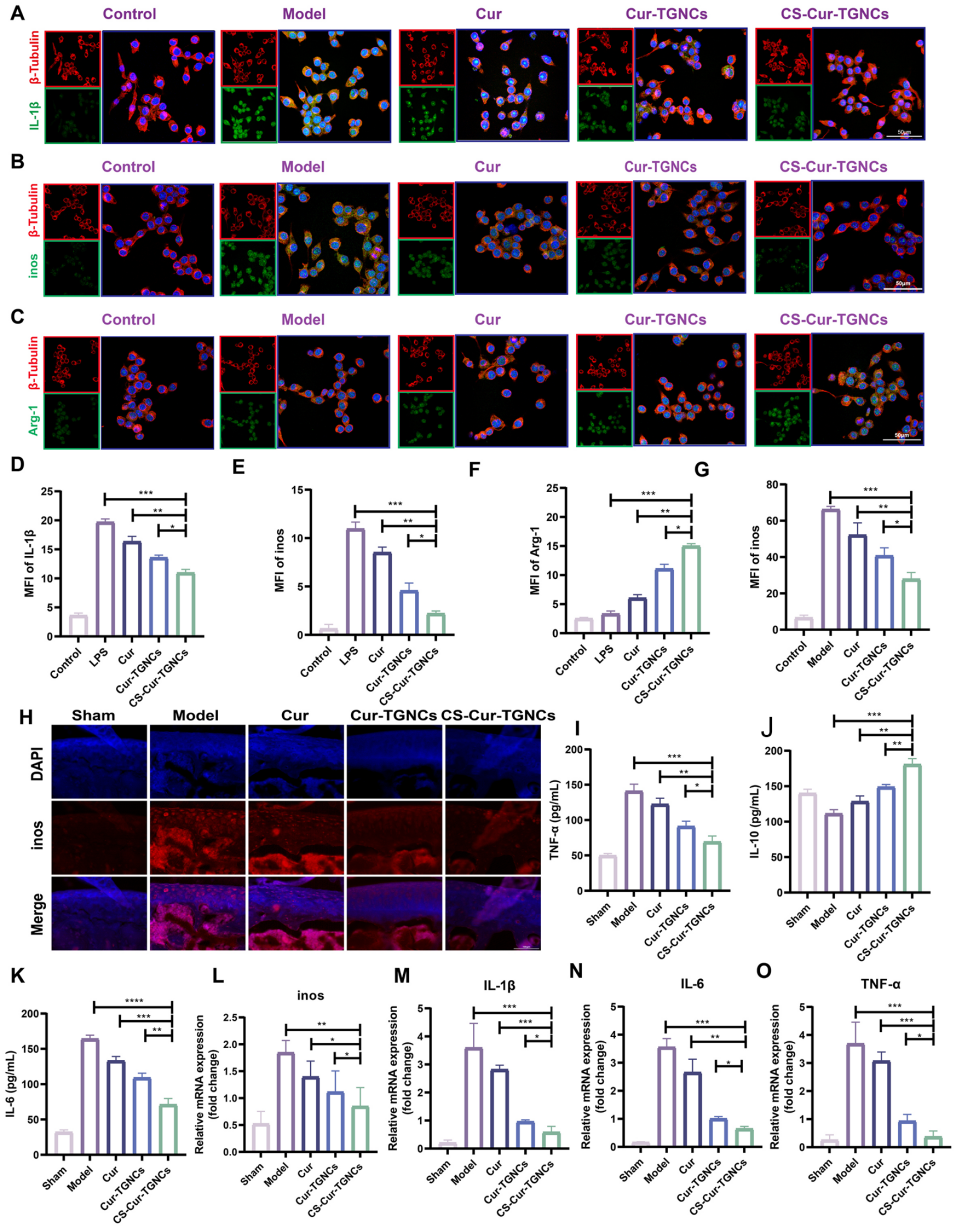
Anti-inflammatory activity of CS-Cur-TGNCs. (**A**, **D**) IL-1β fluorescence images and quantitative analysis of different groups of RAW264.7 cells. (**B**, **E**) iNOS fluorescence images and quantitative analysis of different groups of RAW264.7 cells. (**C**, **F**) Arg-1 fluorescence images and quantitative analysis of different groups of RAW264.7 cells. (**G**, **H**) iNOS fluorescence images and quantitative analysis of different groups of knee joints. (**I**-**K**) Inflammatory factors (TNF-α, IL-10 and IL-6) were detected in the serum of different groups of mice (**L**-**O**) The expression of mRNAs (iNOS, TNF-α, IL-1β and IL-6) in ankle tissues was detected by q-PCR. Data are shown as the mean ± SD (*n* = 3). *P < 0.05, **P < 0.01, ***P < 0.001

To investigate the anti-inflammatory effects of CS-Cur-TGNCs, we evaluated the expression of an inflammatory factor (IL-1β) and the conversion of M1-type macrophages to M2 macrophages after different treatment modalities by immunofluorescence and q-PCR assays. The expression of IL-1β is a key inflammatory factor in arthritic diseases. The cell fluorescence results showed that the expression of IL-1β (green) was significantly increased in LPS-induced RAW264.7 cells. In comparison, the fluorescence intensity gradually decreased after Cur, Cur-TGNCs and CS-Cur-TGNCs treatments (Fig. 5A). Specifically, the fluorescence intensity in the CS-Cur-TGNCs group was approximately 0.63 times greater than that in the Cur-TGNCs group (Fig. 5D), suggesting that the CS-Cur-TGNCs play a significant role in suppressing inflammation. In addition, we examined the expression levels of the M1-type macrophage marker (iNOS) and M2-type macrophage marker by a cytofluorescence assay. The results showed that the M1-type macrophage marker (iNOS) was upregulated by LPS to different degrees after Cur and Cur-TGNCs treatments, and the most significant decrease was observed in the CS-Cur-TGNCs group (Fig. 5B, E). In contrast, the expression of the M2-type macrophage marker Arg-1 was significantly upregulated after CS-Cur-TGNCs treatment and was approximately 2.67-fold and 1.45-fold greater than that in the Cur and Cur-TGNCs groups, respectively (Fig. 5C, F). These results suggest that CS-Cur-TGNCs can promote the transition of M1 macrophages to M2 macrophages. Next, we verified the anti-inflammatory effects of CS-Cur-TGNCs at the animal level by tissue fluorescence, ELISA and q-PCR experiments. As shown in Fig. 5H, both Cur and Cur-TGNCs downregulated the expression of iNOS in the joint area, whereas the CS-Cur-TGNCs-treated group had the lowest fluorescence intensity, which was approximately 0.55-fold and 0.63-fold greater than that of the Cur and Cur-TGNCs groups, respectively (Fig. 5G). Treatment with CS-Cur-TGNCs significantly downregulated the expression of a proinflammatory factor (IL-6 and TNF-α) and upregulated the expression of an anti-inflammatory factor (IL-10) in serum (Fig. 5I-K). q-PCR was used to detect the expression of key inflammatory proteins. Consistent with the tissue fluorescence results, the mRNA expression levels of relevant inflammatory factors (IL-6, iNOS, TNF-α, and IL-1β) were significantly increased in the model mice. Compared with Cur and Cur-TGNCs, CS-Cur-TGNCs inhibited the mRNA expression of inflammatory factors more significantly (Fig. 5L-O). The above experiments demonstrated that CS-Cur-TGNCs have excellent anti-inflammatory effects in the treatment of RA.

### In vivo therapeutic effects on RA

**Fig. 6:**
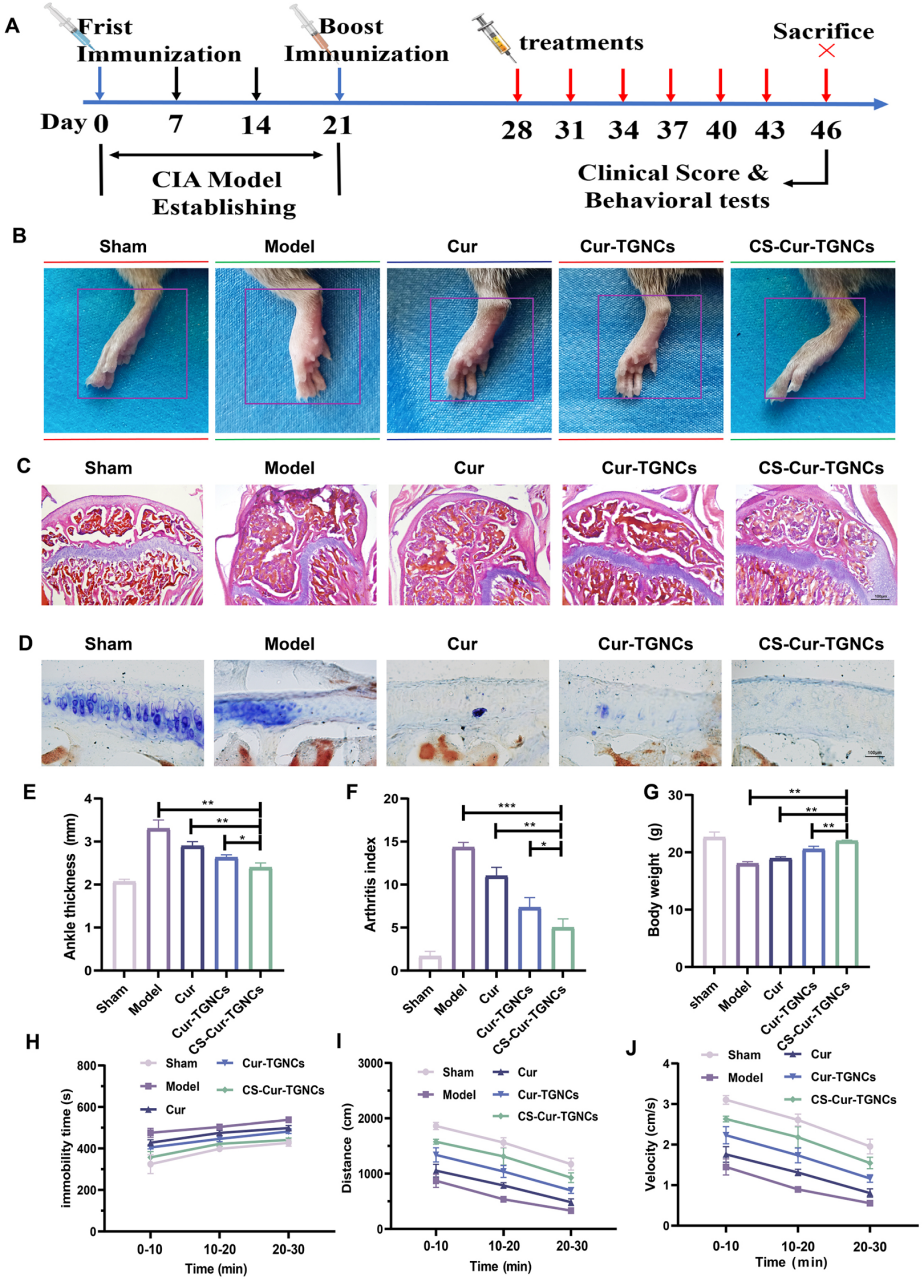
In vivo Therapeutic Effects on RA. (**A**) Establishment of the CIA mouse model and summary of experiments. (**B**) The representative photographs of the posterior ankle joints of different groups of CIA mice. (**C**) H&E images of knee joints from different groups of CIA mice. (**D**) Toluidine blue images of the knee joints of different groups of CIA mice. (**E**-**G**) Changes in hind paw thickness, treatment scores and body weight in different groups of CIA mice. (**H**-**J**) Time at rest, distance traveled, and speed of movement for 30 min in different groups of CIA mice. Data are shown as the mean ± SD (*n* = 3). *P < 0.05, **P < 0.01, ***P < 0.001

We followed the treatment protocol shown in Fig. 6A and recorded joint recovery (photographs, ankle thickness, clinical scores, body weight, exercise recovery, and pathological changes) to explore the therapeutic effects of CS-Cur-TGNCs in CIA mice. Photographs of the joints of CIA mice show marked redness, swelling and articular deformity, with complete swelling of the lateral ankle joints (Fig. 6B). Whereas Cur was able to slightly ameliorate these symptoms, CS-Cur-TGNCs significantly reduced these joint swelling phenomena, showing stronger therapeutic effects than Cur and Cur-TGNCs. Bone erosion and destruction are important pathological features of rheumatoid arthritis for assessing its severity. We performed H&E and toluidine blue staining for histologic analysis of knee joint tissues from different treatment groups. The results showed that the joint tissues of the normal group did not exhibit joint inflammation or cartilage damage, and there was a clear interface between the bone and cartilage tissues. The PBS-treated CIA mice exhibited severe cartilage tissue destruction and severe inflammatory infiltration, but the joint structure was not obvious. Joint inflammation and cartilage erosion were significantly reduced in the Cur and Cur-TGNC groups. Moreover, the efficacy of the CS-Cur-TGNCs treatment was significantly greater than that of the Cur and Cur-TGNCs treatments, but the difference was not significant (Fig. 6C). In addition, the results of toluidine blue staining also showed that more cartilage and proteoglycans were distributed on the joint surface in the CS-Cur-TGNCs-treated group than in the control group (Fig. 6D). These results indicated that CS-Cur-TGNCs had a satisfactory effect on RA treatment. Subsequently, we measured the hind paw thickness, inflammation score and body weight of the mice. Changes in hind paw thickness and inflammation score are important indicators of the therapeutic efficacy of RA. Compared with that in the PBS-treated model group, the hind paw thickness was reduced in the Cur, Cur-TGNCs, and CS-Cur-TGNCs groups. Notably, the CS-Cur-TGNCs-treated group exhibited a more significant difference in cytokine levels (Fig. 6E). Moreover, CS-Cur-TGNCs had the same effect on reducing joint inflammation scores and significantly suppressing joint inflammation in CIA mice (Fig. 6F). Body weight is often used as an indirect indicator of RA recovery. After the injection of CS-Cur-TGNCs, the body weight of the CIA mice significantly increased and almost reached normal values (Fig. 6G). In addition, we evaluated the duration, distance, and movement speed of the mice that were stationary within 30 min in the different treatment groups. The CS-Cur-TGNCs treatment significantly improved the mobility deficits of the CIA mice (Fig. 6H-J). Therefore, we concluded that CS-Cur-TGNCs have a favorable effect on joint recovery.

### In vivo therapeutic effects of GA

**Fig. 7:**
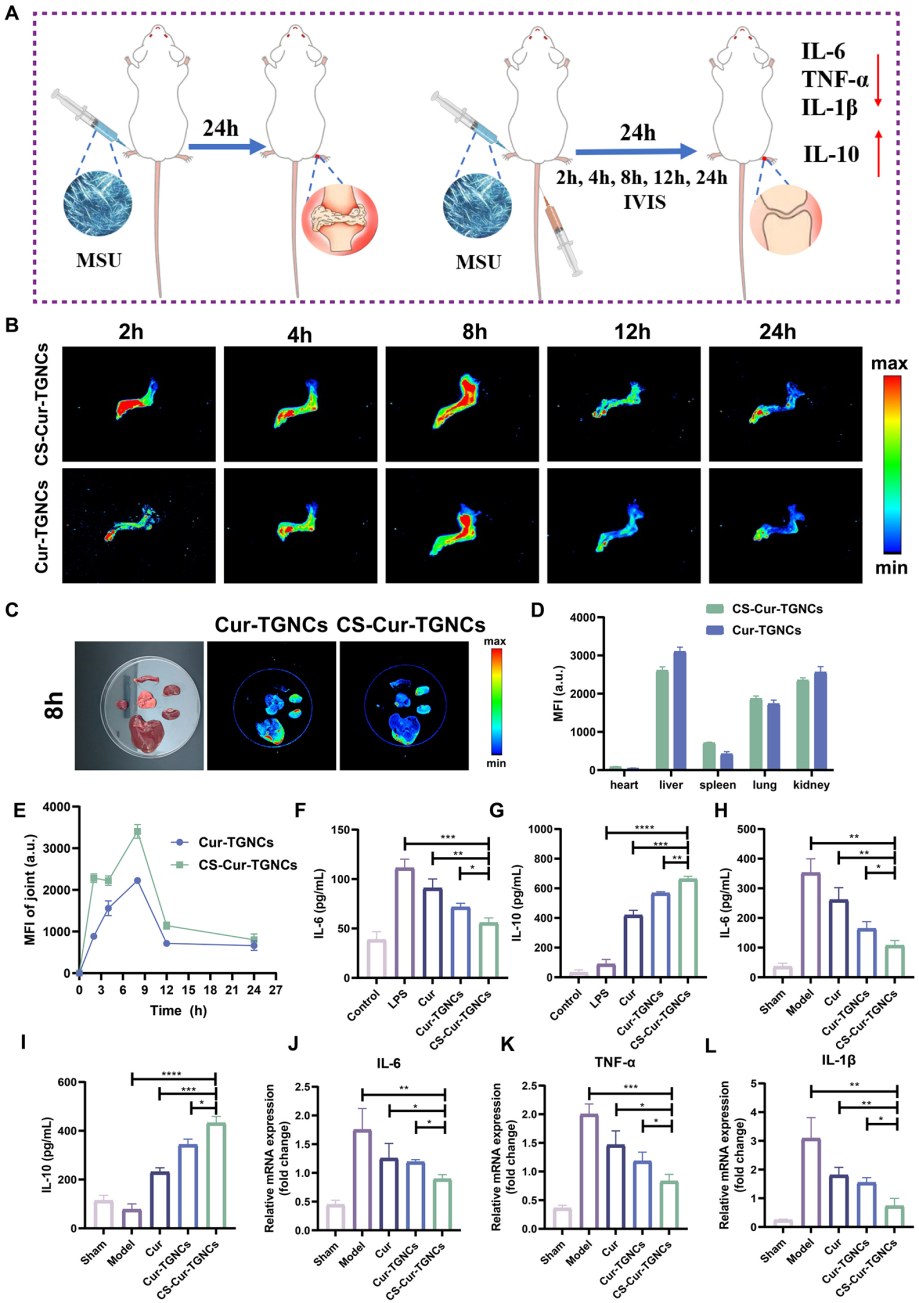
In vivo therapeutic effects of GA. (**A**) Establishment of the GA rat model and summary of experiments. (**B**, **E**) Pictures and statistical analysis of the rat ankle IVIS system. (**C**, **D**) Pictures and statistical analysis of the vital metabolic organs of the rat ankle IVIS system. (**F**-**I**) Inflammatory factors (IL-6, IL-10) were detected in cell supernatants and rat serum of different groups. (**J**-**L**) The expression of mRNAs (TNF-α, IL-1β, and IL-6) in the ankle tissues of GA rats was detected by q-PCR. Data are shown as the mean ± SD (*n* = 3). *P < 0.05, **P < 0.01, ***P < 0.001

To further evaluate the therapeutic effects of CS-Cur-TGNCs on other arthritic diseases, we established a rat GA model according to the scheme shown in Fig. 7A. The joint targeting and inhibition of inflammation by CS-Cur-TGNCs were also evaluated via an in vivo fluorescence imaging system (IVIS), ELISA and q-PCR assays. Figure 7B shows that the fluorescence in the joint tissues of the Cur-TGNC treatment group was weaker than that in the control group, indicating that only a small amount of Cur-TGNCs accumulated at the site of inflammation. Compared with those of Cur-TGNCs, the fluorescence intensity of CS-Cur-TGNCs was greater at different time points (2, 4, 8, 12 and 24 h), and the fluorescence intensity reached a maximum at 8 h. Thereafter, the fluorescence intensity at the joint site gradually weakened with increasing time (Fig. 7E). At 8 h, the fluorescence levels in the liver and kidney of the CS-Cur-TGNCs- and Cur-TGNC-treated groups were significantly greater than those in the other organs (Fig. 7C, D). These results indicated that the CS-Cur-TGNCs and Cur-TGNCs were metabolized mainly by the liver and kidney, which was also consistent with the metabolic characteristics of the majority of the nanoparticles. Next, we assessed the levels of inflammatory factors (IL-6 and IL-10) in cell supernatants and rat serum, as well as the mRNA expression levels of inflammatory factors associated with the ankle joint site (IL-6, TNF-α, and IL-1β), using ELISA and q-PCR. Treatment with CS-Cur-TGNCs significantly downregulated the expression of a proinflammatory factor (IL-6) and upregulated the expression of an anti-inflammatory factor (IL-10) in cell supernatants (Fig. 7F, G). The results for the relevant inflammatory factors in the serum were in good agreement with the above results (Fig. 7H, I). Similar experimental results were obtained by q-PCR. The mRNA expression of proinflammatory factors (IL-6, TNF-α, and IL-1β) in the ankle joint was downregulated in the Cur, Cur-TGNCs, and CS-Cur-TGNCs treatment groups. CS-Cur-TGNCs downregulated the expression of these mRNAs to the most significant extent (Fig. 7J-L). These results suggest that, as a nanomedicine, CS-Cur-TGNCs can be applied not only for the treatment of RA but also for the treatment of GA, as they have excellent joint targeting ability and inflammation.

### CS-Cur-TGNCs promotes joint recovery in GA

**Fig. 8:**
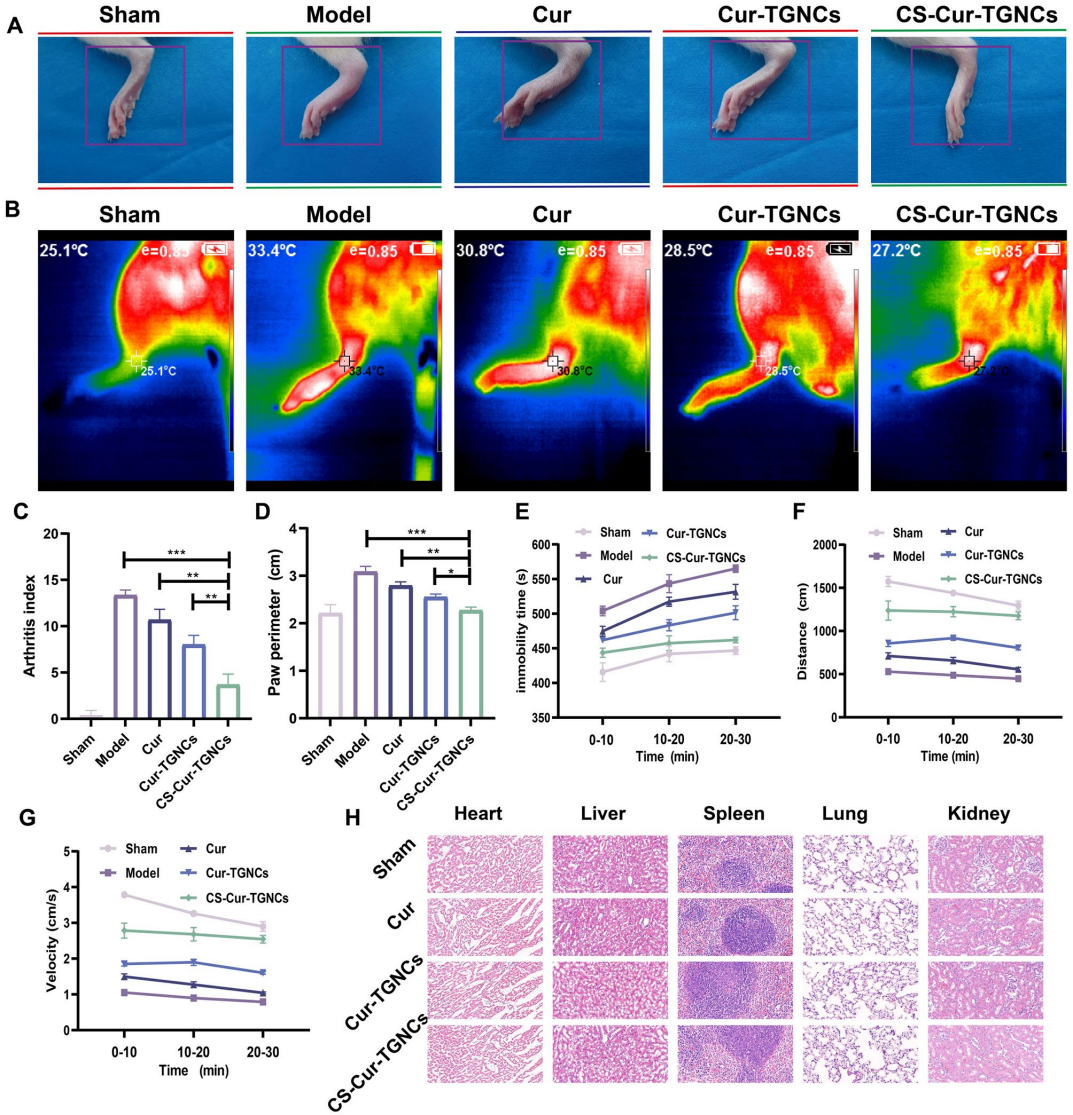
CS-Cur-TGNCs Promotes joint recovery in GA. (**A**) Representative photos of the ankle joint of different groups of rats 24 h after MSU injection. (**B**) The changes of temperature in the ankle joints of different groups of rats. (**C**, **D**) Changes in treatment scores and ankle circumference in different groups of GA rats. (**E**-**G**) Time at rest, distance traveled, and speed of movement for 30 min in different groups of GA rats. (**H**) H&E staining of heart, liver, spleen, lung and kidney sections in each group. Data are shown as the mean ± SD (*n* = 3). *P < 0.05, **P < 0.01, ***P < 0.001

To further investigate the efficacy of CS-Cur-TGNCs on GA, the joint recovery of GA rats was statistically analyzed. We observed that CS-Cur-TGNCs minimized paw swelling and inflammation (Fig. 8A). In addition, inflammation led to an increase in local temperature, and treatment with CS-Cur-TGNCs significantly reduced the local temperature to within the normal range (Fig. 8B). Moreover, CS-Cur-TGNCs exerted superior therapeutic effects on suppressing joint swelling and relieving joint inflammation. The clinical scores of the model rats increased rapidly, and the progression of GA was inhibited to different extents after treatment. Among these combinations, CS-Cur-TGNCs maximally inhibited GA disease progression, resulting in the lowest clinical score (Fig. 8C). Joint swelling was evaluated by measuring the circumference of the ankle joint. Similarly, CS-Cur-TGNCs significantly alleviated joint swelling and inflammation in GA rats (Fig. 8D). We measured the resting time, distance, and movement speed of GA rats in different treatment groups for 30 min. The results also showed that the CS-Cur-TGNCs treatment significantly promoted the recovery of mobility in GA rats (Fig. 8E-G). The biosafety of these nanopreparations is also considered in the treatment of this disease. After 30 days of continuous administration of the different preparations, vital organs, such as the heart, liver, spleen, lung and kidney, were observed via H&E staining. The results showed that there was no significant difference between the different groups (Fig. 8H), indicating that CS-Cur-TGNCs have a good safety profile. The blood of the rat was assayed for blood biochemistry and the results are shown in Fig. S4, which indicates that CS-Cur-TGNCs do not affect the normal functioning of the liver, kidneys and heart. Therefore, we concluded that CS-Cur-TGNCs are safe and effective for the treatment of arthritic diseases.

## Discussion

**Scheme 1:**
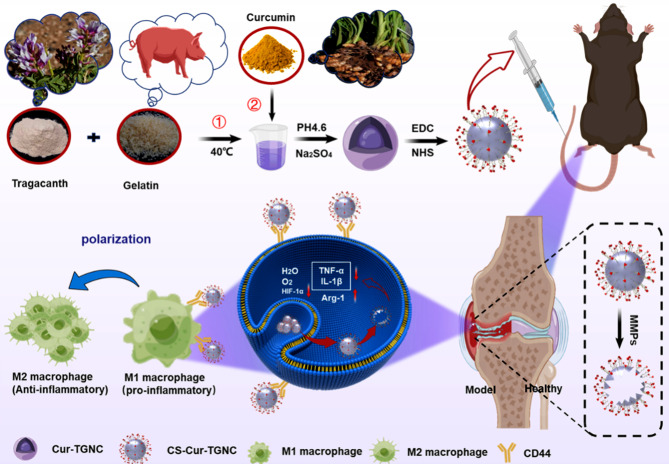
Schematic diagram of the CS-Cur-TGNCs formulation and its mechanism of action in the treatment of arthritis

RA is a highly heterogeneous autoimmune disease characterized by infiltration of synoviocytes and bone erosion in the local joint cavity [40]. The drugs (methotrexate, prednisone and dexamethasone) currently used clinically for the treatment of RA have serious side effects, which greatly limit the use of relevant drugs [41, 42]. As scientific research has progressed, curcumin has been used to treat RA as a natural product with superior anti-inflammatory and antioxidant activity [43]. Cyberpharmacology, based on systems biology and pharmacology, is a powerful method for evaluating the metabolic and efficacy profiles of drugs, analyzing the specific pathways by which drugs treat diseases, and revealing the molecular mechanisms of drug action [44, 45]. The protein‒protein interaction (PPI) network showed that the 36 targets were not independent of each other but had an interactive relationship with each other. In the drug target-disease target network, many target genes can be regulated by Cur, which reflects the multitarget quality of Cur. The enrichment of the 36 target genes was further analyzed via GO and pathway enrichment, and the enriched GO terms were related mainly to the inflammatory response and oxidative stress response (Fig. 3). These findings suggest that Cur can play a therapeutic role in RA by affecting multiple biological processes and signaling pathways to regulate inflammation and oxidative stress. These predictions provide a basis for Cur to act on specific targets for the treatment of RA. However, improving the water solubility and stability of curcumin remains a major challenge [46]. To overcome these problems, nanodelivery systems offer promising solutions. However, chemically synthesized nanoparticles are somewhat immunogenic, making the development of biologically sourced substances as nanodelivery systems crucial [47]. Recently, tragacanth gum and gelatin were shown to have unique bioactivity and safety profiles that enable them to be used as drug delivery vehicles [48, 49]. Inspired by these findings, we designed a targeted drug delivery system (CS-Cur-TGNCs) with antioxidant and anti-inflammatory effects according to Scheme 1. Cur-TGNCs were synthesized by the inborn microcrystallization method using biologically sourced tragacanth gum and gelatin as capsule materials. The antisolvent crystallization method was used to precipitate the Cur in the form of nanocrystals in the solution containing the capsule material. Due to the regulation of pH and the addition of electrolytes, gelatin was preferentially coacervation to the nanocrystal surface based on the high surface energy of the Cur nanocrystals. After formaldehyde crosslinking, the coacervated gelatin formed a closed core-shell structure [50]. In order to improve the targeting properties, chondroitin sulfate was used to modify the Cur-TGNCs. As a core-shell structure for nanopreparation, CS-Cur-TGNCs have the advantages of improved Cur stability and water solubility, site-directed release of drugs, and targeting of inflammatory joint sites. An increase in the stability and aqueous solubility of Cur improves its bioavailability, enhancing its therapeutic efficacy (Fig. 1). Because of the high affinity of CS for the CD44 receptor, CS-Cur-TGNCs showed excellent targeting activity to inflammatory joint sites. It can effectively reduce the loss of Cur during circulation in vivo and promote its massive accumulation at disease sites. Moreover, the high expression of matrix metalloproteinases in the inflammatory sites can efficiently degrade the gelatin components of CS-Cur-TGNCs and achieve the site-directed release of Cur at the inflammatory site, which further enhances the therapeutic efficacy of the drug (Figs. 1 and 2). Combined with the results of the CCK-8, hemolysis and H&E experiments, these findings showed that the CS-Cur-TGNCs had good biosafety (Figs. 1K, L and M and 8H), which demonstrated their suitability as drug delivery carriers for the treatment of joint inflammation.

Based on the predicted network pharmacology results, CS-Cur-TGNCs exerted superior therapeutic effects on oxidative stress and inflammation in RA. Oxidative stress (OS) is involved in the pathogenesis of RA and has a strong influence on its pathogenesis [51]. OS is a state in which the oxidative system exceeds the antioxidant system, causing an imbalance between the two. This imbalance not only causes lipid peroxidation and DNA oxidative damage but also causes abnormalities in cellular signal transduction pathways [52]. Oxidative damage in RA is caused mainly by reactive oxygen species (ROS), which destroy hyaluronic acid in synovial fluid, inhibit cartilage matrix production, and cause cartilage degradation, ultimately resulting in damage to articular cartilage [53]. Oxidative markers such as malondialdehyde (MDA), superoxide dismutase (SOD) and glutathione (GSH) are present in RA patients [54]. Changes in the levels of these substances also reflect the level of oxidative stress in the body. Moreover, CS-Cur-TGNCs not only exhibited in vitro antioxidant activity (Fig. 4A, B) but also effectively inhibited oxidative stress-related MDA accumulation, increased GSH and SOD levels (Fig. 4C, D, E), and protected joint tissues. In addition, hypoxia plays an important role in the pathogenesis of RA. Hypoxia at the disease site results in the upregulation of hypoxia-inducible factor-1α (HIF-1α) expression and the massive production of reactive oxygen species (ROS), which can ultimately lead to damage joint tissues. Notably, CS-Cur-TGNCs were also able to ameliorate the hypoxic state of diseased tissues and reduce ROS levels (Fig. 4F, G, H and I).

Macrophages are the most abundant type of immune cell at local inflammatory sites in RA, and macrophages can polarize into proinflammatory (M1) and anti-inflammatory (M2) phenotypes under different tissue microenvironments and pathological conditions [55]. M1 macrophages are found mainly in the early stage of inflammation and can promote joint inflammation by secreting inflammatory cytokines such as TNF-α, IL-1β and IL-6^56^. In contrast, M2 macrophages secrete mainly anti-inflammatory cytokines, such as IL-10 and Arg-1, which play important roles in reducing inflammation and tissue repair [57]. In the joints of RA patients, macrophages are predominantly polarized to the M1 subtype due to the specific inflammatory milieu, so altering macrophage polarization toward the M2 phenotype is an effective treatment for alleviating RA symptoms [58]. Moreover, the CS-Cur-TGNCs were able to release Cur at specific inflammatory sites, thus effectively controlling the development of inflammation. In an in vitro cellular assay, CS-Cur-TGNCs significantly reduced the expression of IL-1β in LPS-stimulated RAW264.7 cells, as observed by immunofluorescence (Fig. 5A, D), confirming the anti-inflammatory effect of CS-Cur-TGNCs. Furthermore, CS-Cur-TGNCs significantly decreased the expression of the M1 marker inos and increased the expression of the M2 marker Arg-1 (Fig. 5B, C, E, and F), suggesting that CS-Cur-TGNCs can promote the conversion of RAW264.7 cells to M2 macrophages, which could reduce inflammation and exert an anti-inflammatory effect. Subsequently, the anti-inflammatory effect of CS-Cur-TGNCs was further confirmed in animal experiments. Tissue immunofluorescence staining revealed a significant reduction in the number of inos at the injury site after CS-Cur-TGNCs treatment (Fig. 5G, H). The q-PCR confirmed that CS-Cur-TGNCs could reduce the expression of inflammation-related mRNAs (inos, TNF-α, IL-6 and IL-1β) (Fig. 5I-L). Behavioral assessments allowed for a more intuitive examination of the therapeutic effects of CS-Cur-TGNCs. Moreover, CS-Cur-TGNCs effectively reduced RA joint swelling, promoted motor recovery and effectively protected joint self-tissues (Fig. 6). These results demonstrated that CS-Cur-TGNCs have an obvious anti-inflammatory effect and can effectively inhibit the development of inflammation at RA joints (Fig. 4). Based on the therapeutic mechanism of CS-Cur-TGNCs in the treatment of RA, further exploration of its therapeutic role in other joint diseases is highly meaningful. We selected gouty arthritis patients to further validate the joint therapeutic effects of CS-Cur-TGNCs. Gouty arthritis (GA) is a nonspecific inflammatory response disease that occurs as a result of the deposition of urate crystals in joints and surrounding soft tissues and has many characteristics [59], such as rapid onset and recurrent attacks over time. In GA, a large number of inflammatory factors (TNF-α, IL-6, and IL-1β) are released due to the massive infiltration of immune cells, which in turn exacerbates joint damage and further exacerbates the extent of inflammation. Therefore, rapid elimination of the immune–inflammatory cascade response, inhibition of inflammatory mediator production, and rapid alleviation of clinical symptoms are the main therapeutic strategies for treating GA [60, 61]. As a nanodrug delivery system targeting CD44, CS-Cur-TGNCs accumulated at the junction site of GA (Fig. 7B, E). At sites of joint inflammation, CS-Cur-TGNCs can be degraded by matrix metalloproteinases, leading to the rapid release of Cur, which is essential for rapid suppression of the inflammatory response. The ELISA results confirmed that CS-Cur-TGNCs inhibited the production of macrophage-related anti-inflammatory factors and reduced the inflammatory response (Fig. 7F-I). The q-PCR results further demonstrated the inhibitory effect of CS-Cur-TGNCs on inflammation in GA joints (Fig. 7J-L). We concluded that the anti-inflammatory effect of CS-Cur-TGNCs contributed to the recovery of motor function in GA rats. A series of behavioral assessments of GA rats showed that CS-Cur-TGNCs effectively suppressed joint swelling, reduced the temperature of diseased joints, and promoted the recovery of motor function (Fig. 8A-G). Undoubtedly, CS-Cur-TGNCs, as a novel nanomedicine, provide a new reference for the treatment of arthritic diseases such as RA and GA.

## Conclusion

In the present study, chondroitin sulfate-modified tragacanth gum–gelatin composite nanocapsules loaded with curcumin nanocrystals were successfully prepared. Moreover, CS-Cur-TGNCs have a good biosafety profile and can significantly improve the stability, water solubility and bioavailability of Cur. Their anti-inflammatory, anti-rheumatoid factor and joint-protective effects have been demonstrated in vitro at the cellular and in vivo animal levels and are effective in the treatment of rheumatoid arthritis. In the treatment of gouty arthritis (GA), joint inflammation was significantly suppressed, and joint tissue was significantly restored in GA rats after CS-Cur-TGNC treatment. Therefore, we believe that the use of CS-Cur-TGNCs as a drug delivery platform for arthritis-related diseases has broad application prospects and could lead to new insights into the clinical treatment of these diseases.

### Electronic supplementary material

Below is the link to the electronic supplementary material.

Supplementary Material 1

## Data Availability

No datasets were generated or analysed during the current study.

## Abbreviations

RA: Rheumatoid arthritis
GA: Gouty arthritis
Cur: Curcumin
CS: Chondroitin sulfate
TGNCs: Tragacanth gum–gelatin Composite nanocapsules
Cur-NCs: Curcumin nanocrystals
Cur-TGNCs: Tragacanth gum–gelatin composite nanocapsules loaded with urcumin nanocrystals
CS-Cur-TGNCs: Chondroitin sulfate-modified tragacanth gum–gelatin composite nanocapsules loaded with curcumin nanocrystals
MMPs: Matrix metalloproteinases
PM: Physical mixture of Cur, tragacanth gum and gelatin
