# Supplementary material for: Chondroitin sulfate-modified tragacanth gum–gelatin composite nanocapsules loaded with curcumin nanocrystals for the treatment of arthritis
Source: J Nanobiotechnology. 2024 May 20;22:270. doi: 10.1186/s12951-024-02540-2 (PMC11104008; doi:10.1186/s12951-024-02540-2)
Supplement: Supplementary file 1 — Supplementary Material 1 [file 12951_2024_2540_MOESM1_ESM.docx]

**Chondroitin sulfate-modified** **tragacanth gum–gelatin composite nanocapsules loaded with curcumin nanocrystals for the treatment of arthritis**

Junpeng Sun ^a, b^, Jiaqun Du ^a, b^, Xiaobang Liu ^a, b^, Jinyu An ^a, b^, Yu Hu ^b, c^, Jing Wang ^a, b^, Fu Zhu ^a, b^, Huicong Feng ^b, c^, Shuai Cheng ^b, c^,He Tian ^b, c *^, Xifan Mei ^b, d, e *^, Chao Wu ^a, b, e*^

^a^ Pharmacy School, Jinzhou Medical University, Jinzhou, Liaoning 121001, China

^b^ Liaoning Provincial Collaborative Innovation Center of Medical Testing and Drug Development, Jinzhou Medical University, Jinzhou, Liaoning 121001, C

^c^ School of Basic Medicine, Jinzhou Medical University, Jinzhou, Liaoning 121001, China.

^d^ The Third Affiliated Hospital of Jinzhou Medical University, Jinzhou, Liaoning 121001, China

^e^ Liaoning Provincial Key Laboratory of Medical Tissue Engineering, Jinzhou Medical University, Jinzhou, Liaoning, 121001, China

*Corresponding author:

Prof. Chao Wu

E-mail: wuchao@jzmu.edu.cn

Address: Pharmacy School, Jinzhou Medical University, Jinzhou, Liaoning 121001, China

Prof. He Tian

E-mail: tianhe@jzmu.edu.cn

Address: School of Basic Medicine, Jinzhou Medical University, Jinzhou, Liaoning 121001, China.

Dr. Xifan Mei

E-mail: meixifan@jzmu.edu.cn

Address: The Third Affiliated Hospital of Jinzhou Medical University, Jinzhou, Liaoning 121001, China

Authors E-mail address:

Junpeng Sun: sjp783686581@163.com

Jiaqun Du: djq1808@163.com

Xiaobang Liu: lxb1025431521@163.com

Jinyu An: anjinyuuu@163.com

Yu Hu: huyu980908@163.com

Jing Wang: 15641656541@163.com

Fu Zhu：[15241156669@163.com](mailto:15241156669@163.com)

Huicong Feng: 13234040869@163.com

Shuai Cheng: [cscs@stu.jzmu.edu.cn](mailto:cscs@stu.jzmu.edu.cn)

**Supporting methods**

**1. Study of Cur stability**

Equivalent CS-Cur-TGNCs and Cur were dissolved in PBS (pH=7.4) to observe the water solubility of the preparations. Cur and CS-Cur-TGNCs were incubated at a final fixed concentration of 40 μM (Cur) in a 150 rpm/min rotary shaker at 37 °C for 6 h. At pre-designed time points (1 h-6 h), 1 mL of the solution was taken from the total solution and the Cur content was determined as described above.

CS-Cur-TGNCs was dispersed in different buffers (water, PBS (PH=7.4), 0.9% Nacl, DMEM, and 10% FBS) and allowed to stand for 7 d at room temperature. After resuspension, the changes in the dispersion state and the stability of CS-Cur-TGNCs were observed.

**2. Hemolytic test**

Hemolysis assay was used to test the biosafety of CS-Cur-TGNCs. Fresh blood from mice was collected and washed three times with PBS and centrifuged to separate erythrocytes. Saline was added to obtain 2% (V/V) erythrocyte solution, and CS-Cur-TGNCs (125, 250, 375, 500, 750, and 1000 μg/mL) was incubated (37 °C) with the same volume of erythrocyte solution for 3 h. The supernatant was collected by centrifugation for 15 min at 1500 rpm. Saline was used as a negative control and water as a positive control. The absorbance of the supernatant was measured at 516 nm using a microplate reader (Versa Max, Molecular Devices, Sunnyvale, CA, USA) and the hemolysis rate was calculated.

Hemolysis rate = (absorbance value of sample - absorbance value of negative control) / (absorbance value of positive control - absorbance value of negative control) × 100%.

**4. In vitro release studies of CS-Cur-TGNCs**

The release behavior of the drug was examined using a shaker (SHZ-82, Jintan Science Analysis Instrument Co., Ltd., Jiangsu, China). 0.3% (V/V) Tween-80 was used as the release medium. and in the presence or absence of mmp-2 (2.5 μg/mL) in the release medium, the sample (Cur; 2.5 mg) was added to the release medium (300 mL, 37 °C). At the set time point, at the same liquid level, the release medium (2 mL) was collected and filtered. Subsequently, the same volume of release medium was injected into the system to constant the dissolution volume. Finally, the concentration of Cur was determined using a UV spectrophotometer (UV-757CRT, Shanghai Precision Scientific Instrument Co., Ltd. Shanghai, China) at 425 nm and the release rate was calculated.

**3. Cell cytotoxicity in vitro**

Cytotoxicity of CS-Cur-TGNCs on RAW264.7 cells was determined using CC-K8 kit. Cell suspensions of RAW264.7 were inoculated in 96-well plates (100 μL, density 5000/well) and 3-well replicates were done and incubated overnight. Subsequently, different concentrations of CS-Cur-TGNCs (0.05, 0.25, 0.5, 2.5, 5, 10, 40, 80, 160, 320 μg/mL) were added to the 96-well plate and incubated for 4 h. After that, CC-K8 reagent (10 μL/well) was added to the 96-well plate, and the plate was incubated for 4 h. OD values were measured at 450 nm using a microplate reader (Versa Max, Molecular Devices, Sunnyvale, CA, USA). The cell viability was calculated as follows:

cell viability (%) = ODt / ODc×100%

ODt represents the absorbance of the treated cells, ODc represents the absorbance of the control cells.

**4. Network pharmacological study of the mechanism of action of curcumin in the treatment of rheumatoid arthritis**

The CAS of curcumin was determined to be 458-37-7, with a molecular weight of 368.38. The 2D results for curcumin were obtained from the PubChem database, and uploaded to the Swiss Target Prediction database for analysis for curcumin target prediction. Subsequently, the GeneCards database was accessed, and the RA disease targets were collected using "Rheumatoid Arthritis" as the keyword. The obtained curcumin targets and RA disease targets were subsequently sorted and counted, and the intersecting targets were screened to construct a Wayne diagram using an online mapping tool. Wein diagrams were drawn using an online mapping tool. The shared gene targets were uploaded into the String database, and a protein‒protein interaction (PPI) network was constructed to screen out the core rake points of curcumin for the treatment of RA. Finally, GO (Gene Ontology) biofunctional annotation and KEGG enrichment analyses of the intersecting core targets were performed using the DAVID database, and correlation analyses were performed on the target biofunctions and the pathways involved.

**5. Pharmacokinetic study in SD rats**

The pharmacokinetics (PK) of CS-Cur-TGNCs were studied in Sprague‒Dawley (SD) rats. Six SD rats were randomized into three groups (n=3). Cur and CS-Cur-TGNCs (10 mg/kg of Cur) were injected intravenously. Whole blood was collected from the fundus venosus at predetermined time points (5 min, 15 min, 45 min, 1 h, 2 h, 4 h, 6 h, 8 h, 12 h, and 24 h) and centrifuged at 3000 rpm to obtain plasma (200 μL). Nitradipine (2 mg/mL, 10 μL) was added to 150 μL of plasma as an internal standard, vortexed and mixed for 30 s, and 500 μL of ethyl acetate was added; the mixture was vortexed for 3 min. The samples were centrifuged at 3500 rpm for 5 min at room temperature, after which the supernatant was removed. Then, 500 μL of ethyl acetate was added to the precipitate, which was extracted again. The two supernatants were combined and blown dry under nitrogen. The residue was added to 100 μL of the mobile phase, vortexed and mixed for 3 min and centrifuged at 10000 rpm for 15 min, after which 50 μL of the supernatant was collected and analyzed via high-performance liquid chromatography (HPLC; Shimadzu LC-2030). Acetonitrile and 5% glacial acetic acid (55:45) were used as the mobile phase, the detection wavelength was 430 nm, and the column temperature was 35 °C. Pharmacokinetic parameters were calculated and analyzed using Pksolver version 2.0.


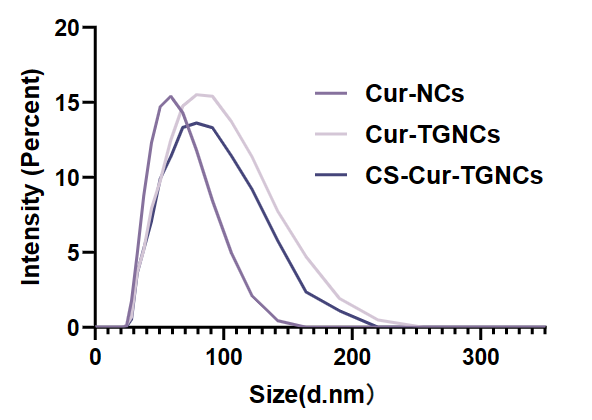
**Supporting results**

Fig. S1. The particle size distribution of Cur-NCs, Cur-TGNCs and CS-Cur-TGNCs.


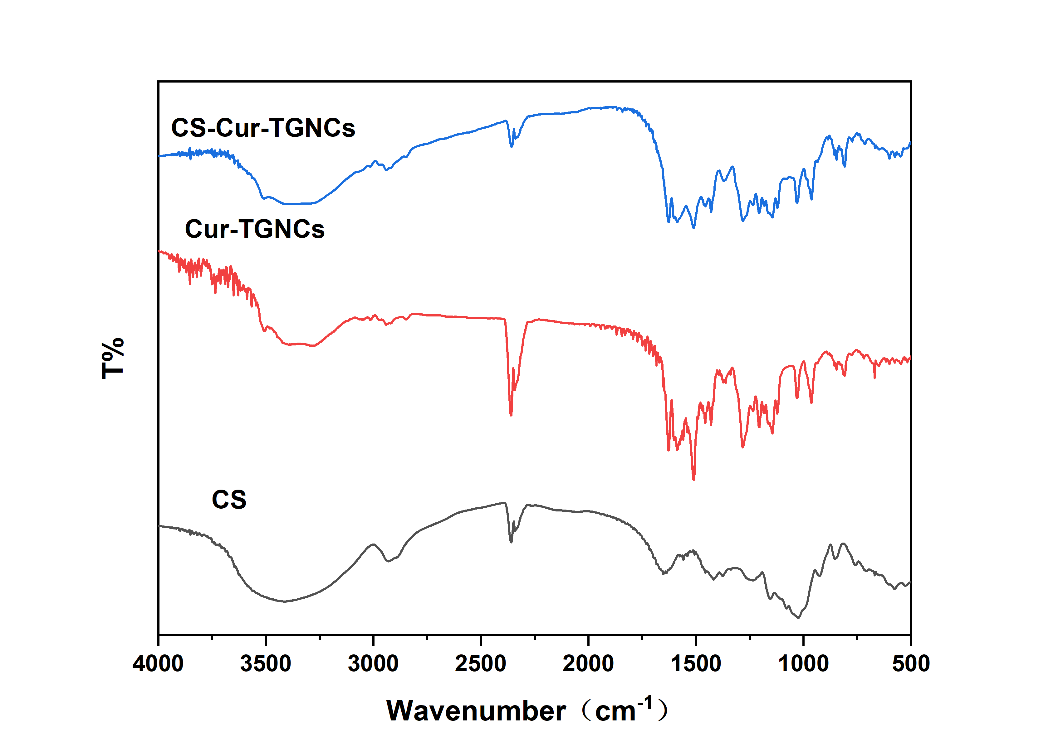
Fig. S2. FTIR spectra of CS, Cur-TGNCs and CS-Cur-TGNCs.


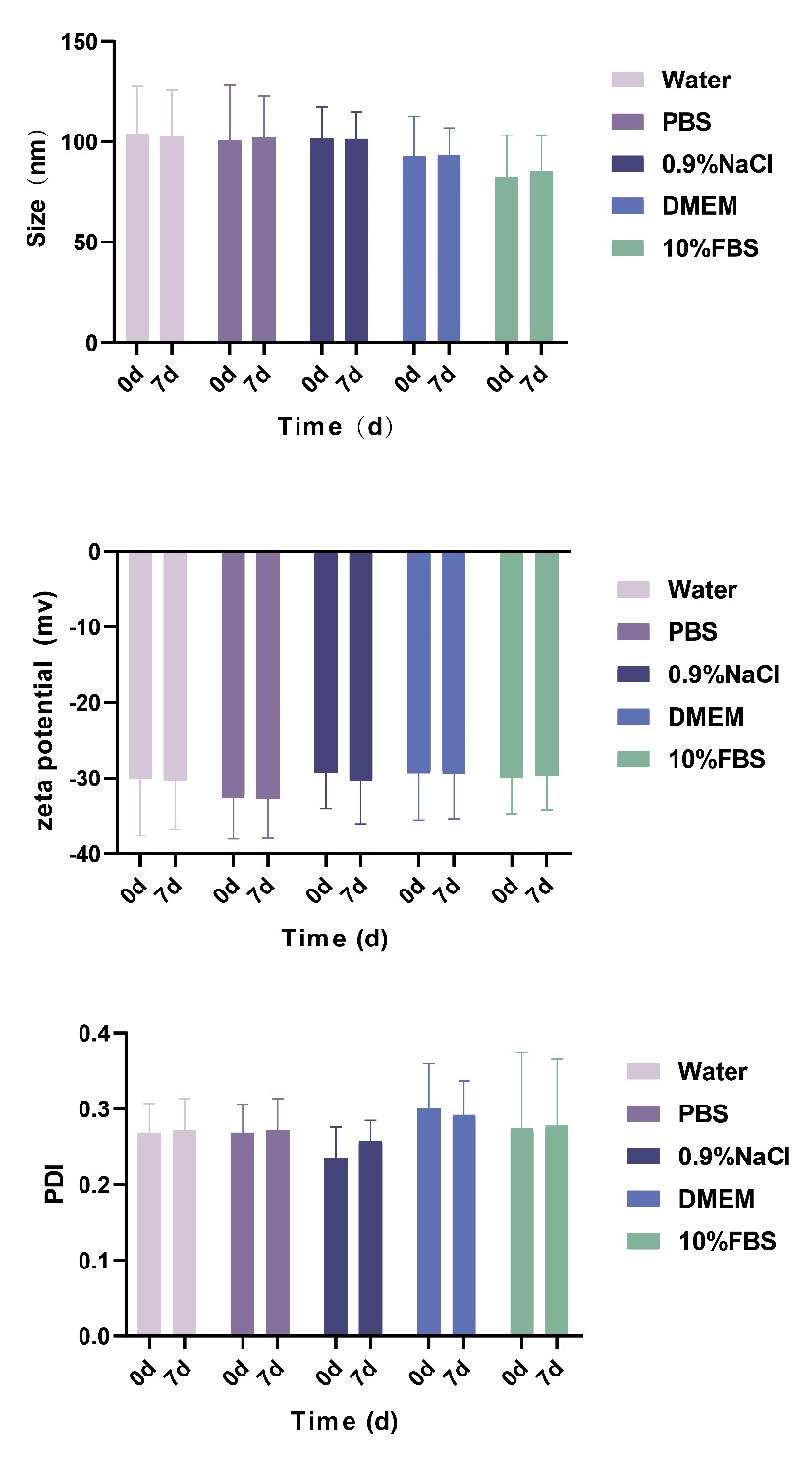


Fig. S3. The changes in particle size, ZP and PDI values of CS-Cur-TGNCs at 0 d and 7 d in different media.


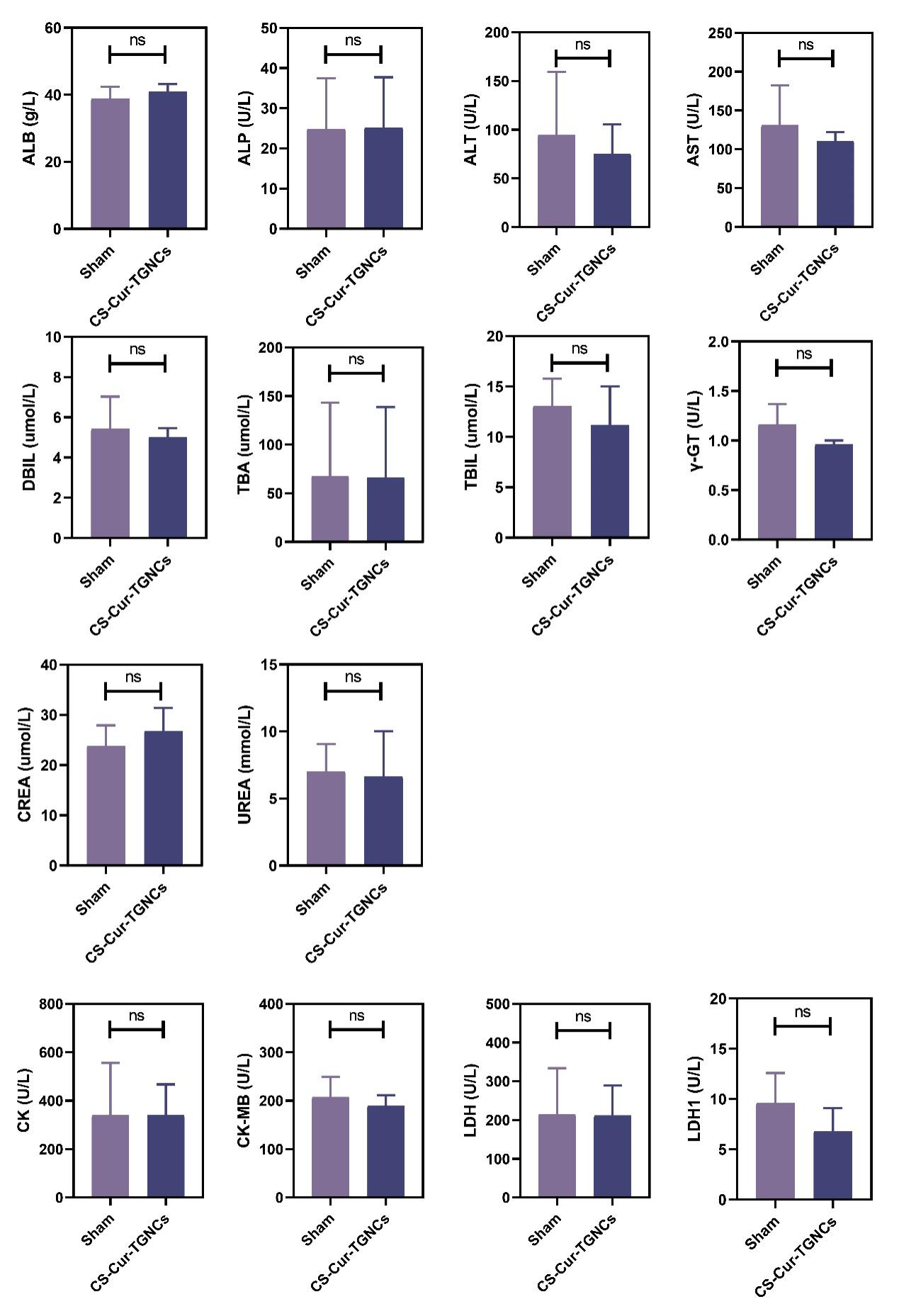


Fig. S4. Blood biochemical assays in rat.
